# Mononuclear-macrophages but not neutrophils act as major infiltrating anti-leptospiral phagocytes during leptospirosis

**DOI:** 10.1371/journal.pone.0181014

**Published:** 2017-07-11

**Authors:** Xu Chen, Shi-Jun Li, David M. Ojcius, Ai-Hua Sun, Wei-Lin Hu, Xu’ai Lin, Jie Yan

**Affiliations:** 1 Collaborative Innovation Center for Diagnosis and Treatment of Infectious Diseases, Zhejiang University, Hangzhou, Zhejiang, P.R. China; 2 Department of Medical Microbiology and Parasitology, Zhejiang University School of Medicine, Hangzhou, Zhejiang, P.R. China; 3 Division of Basic Medical Microbiology, State Key Laboratory for Diagnosis and Treatment of Infectious Diseases, the First Affiliated Hospital, Zhejiang University School of Medicine, Hangzhou, Zhejiang, P.R. China; 4 Guizhou Provincial Center for Disease Control and Prevention, Guiyang, Guizhou, P.R. China; 5 Department of Biomedical Sciences, University of the Pacific, Arthur Dugoni School of Dentistry, San Francisco, California, United States of America; 6 Faculty of Basic Medicine, Hangzhou Medical College, Hangzhou, Zhejiang, P.R. China; Cornell University, UNITED STATES

## Abstract

**Objective:**

To identify the major infiltrating phagocytes during leptospirosis and examine the killing mechanism used by the host to eliminate *Leptospira interrogans*.

**Methods:**

Major infiltrating phagocytes in *Leptospira*-infected C3H/HeJ mice were detected by immunohistochemistry. Chemokines and vascular endothelial cell adhesion molecules (VECAMs) of *Leptospira*-infected mice and leptospirosis patients were detected by microarray and immunohistochemistry. *Leptospira*-phagocytosing and -killing abilities of human or mouse macrophages and neutrophils, and the roles of intracellular ROS, NO and [Ca^2+^]i in *Leptospira*-killing process were evaluated by confocal microscopy and spectrofluorimetry.

**Results:**

Peripheral blood mononuclear-macrophages rather than neutrophils were the main infiltrating phagocytes in the lungs, liver and kidneys of infected mice. Levels of macrophage- but not neutrophil-specific chemokines and VECAMs were significantly increased in the samples from infected mice and patients. All macrophages tested had a higher ability than neutrophils to phagocytose and kill leptospires. Higher ROS and NO levels and [Ca^2+^]i in the macrophages were involved in killing leptospires. Human macrophages displayed more phagolysosome formation and a stronger leptospire-killing ability to than mouse macrophages.

**Conclusions:**

Mononuclear-macrophages but not neutrophils represent the main infiltrating and anti-leptospiral phagocytes during leptospirosis. A lower level of phagosome-lysosome fusion may be responsible for the lower *Leptospira*-killing ability of human macrophages.

## Introduction

Leptospirosis is a global zoonotic infectious disease caused by pathogenic *Leptospira* species [1]. The disease is endemic in Asia, South America, and Oceania [2–5]. Moreover, in recent years, human leptospirosis has been considered as an emerging infectious disease in Europe and North America [6–8]. Over one million human leptospirosis cases have been reported annually, and the mortality rate ranges from 5 to 20% worldwide [9].

Many animals, such as livestock and rodents, serve as the hosts of pathogenic *Leptospira* species, and most the infected animals continuously discharge leptospires through their urine to contaminate soil and water [10]. Human individuals can be infected when they come in contact with *Leptospira*-contaminated soil or water [1,11]. After pathogenic leptospires invade into the human body through mucosa or abrasive skin, the spirochetes rapidly enter the bloodstream to cause septicemia and then diffuse into internal organs or tissues, such as lungs, liver and kidneys, to cause pathological injury [12]. Human leptospirosis is present in an extremely broad clinical spectrum ranging from mild influenza-like illness to severe life-threatening forms characterized by high fever, myalgia and jaundice, to pulmonary diffuse hemorrhage, meningitis and renal failure [10–12].

Phagocytosis is one of the main mechanisms to eliminate invading microbial pathogens in early stages of infection in individuals without acquired anti-infection immunity [13]. Macrophages and neutrophils are the major phagocytes responsible for killing and elimination of many invasive pathogens [14]. In the infected tissues and organs, macrophages and neutrophils are also the main infiltrating cells during acute bacterial infection [13,14]. The two types of phagocytes phagocytose pathogens by first ingesting them into phagosomes, then fusing phagosomes with lysosomes to form phagolysosomes, and finally killing and degrading the pathogens through the phagolysosome’s acidic environment, reactive oxygen species (ROS), nitric oxide (NO) and hydrolases [15]. Moreover, the intracellular free calcium ion concentration ([Ca^2+^]i) also plays an important role in the pathogen-killing process, such as promoting fusion between phagosomes and lysosomes, causing a respiratory burst for ROS production, and inducing secretion of microbicidal granules in macrophages and neutrophils [16]. However, many different types of phagocytes infiltrate tissues in infectious diseases caused by different pathogens. For example, pathogenic cocci cause pyogenic infection due to infiltration of neutrophils [17], while many pathogens belonging to the genus of *Salmonella* usually cause nonpyogenic infection in which mononuclear-macrophages but not neutrophils act as the main infiltrating phagocyte [18]. However, whether mononuclear-macrophages or neutrophils from peripheral blood represent the main infiltrating anti-leptospiral phagocyte in infected tissues during leptospirosis has not been reported yet.

Infiltration of mononuclear-macrophages or neutrophils during infection is a migratory process of these phagocytes from the peripheral blood towards the infected tissues that involves adhesive and chemotactic steps [19]. Specific chemokines and vascular endothelial cell adhesion molecules (VECAMs) induce this migration of phagocytes [20]. Monocyte chemoattractant protein (MCP), macrophage inflammatory protein (MIP) and vascular cell adhesion molecule (VCAM) but human interleukin-8 (IL-8), mouse keratinocyte-derived chemokine (KC) and intercellular adhesion molecule (ICAM) have been confirmed as the major chemokines and VECAMs for mononuclear-macrophages and neutrophils, respectively, while E-selectin and P-selectin contribute to the adhesion for both the two types of phagocytes [19,20]. Therefore, the levels of these factors affect the types of phagocytes that infiltrate during infection. However, the chemokines and VECAMs involved in migration of phagocytes in leptospirosis are poorly understood.

*L*. *interrogans* is the most prevalent pathogenic *Leptospira* species in the world [1,10]. Although many serogroups and serovars of *L*. *interrogans* are present in China, *L*. *interrogans* serogroup Icterohaemorrhagiae serovar Lai is responsible for disease in over 60% of Chinese leptospirosis patients [2,11]. In the present study, we therefore investigated the types of infiltrating phagocytes in lungs, liver and kidneys, and the profiles of chemokines and VECAMs during infection of *L*. *interrogans* strain Lai, and the mechanisms used by mononuclear-macrophages and neutrophils to kill the spirochetes. This study confirmed that mononuclear-macrophages, but not neutrophils, are the main infiltrating cells responsible for elimination of *L*. *interrogans* during infection.

## Materials and methods

### Ethics statement

All subjects gave written informed consent, and the study was approved by the Human Ethics Committee of the Medical School of Zhejiang University, and complied with the Declaration of Helsinki. All animal experiments were performed in strict accordance with the National Regulations for the Administration of Experimental Animals of China (1988–002) and the National Guidelines for Experimental Animal Welfare of China (2006–398). All the animal experimental protocols were approved by the Ethics Committee for Animal Experiments of Zhejiang University.

### Leptospiral strain and culture

*L*. *interrogans* serogroup Icterohaemorrhagiae serovar Lai strain Lai was provided by the Chinese National Institute for Control of Pharmaceutical and Biological Products in Beijing, China. The leptospiral strain was cultivated at 28°C in Ellinghausen-McCullough-Johnson- Harris (EMJH) liquid medium [21].

### Animals

Female C3H/HeJ mice were provided by the Laboratory Animal Center of Zhejiang University (Certificate No.: SCXK[zhe]2007–0030). They were 15±1 g, three weeks old, for experiments with *Leptospira*-infected mice; and 20 ± 2 g, six weeks old, for separation of peripheral blood monocytes and neutrophils. All the animals in the subsequent experiments were euthanized by CO_2_ inhalation.

### Isolation and differentiation of primary human or mouse monocytes

Human or mouse peripheral blood monocytes (Hu- or Ms-monocytes) were isolated from healthy volunteers or from C3H/HeJ mice using human or mouse MicroBeads monocyte separation kit (Miltenyi Biotec, Germany) according to the manufacturer’s protocol. Briefly, peripheral blood mononuclear cells (PBMCs) in EDTA-anticoagulated human or murine peripheral blood samples were isolated on a 400×g Ficoll-Paque gradient centrifugation at room temperature for 30 min. The collected PBMCs were suspended in 10 mM phosphate buffered saline (PBS, pH7.4) and then filtered with a 40 μm cell strainer. After washing twice with PBS, the PBMCs were mixed with human CD14 or mouse CD11b magnetic beads for a 15-min incubation at 4°C and then running through a magnetic LS column. The purity (>95%) of isolated Hu- or Ms-monocytes was determined by flow cytometry (type FACSCalibur, Beckman Coulter, USA) using human monocyte surface marker CD14-APC or mouse monocyte surface maker CD11b-APC (eBioscience, USA). In addition, the possible contamination of neutrophils and eosinophils in the isolated Hu- or Ms-monocytes was also tested by flow cytometry using FITC-labeled mouse anti-human neutrophil surface marker CD15-IgG or rat anti-mouse neutrophil surface marker Ly6G-IgG (BD Biosciences, USA) and FITC- or BB515-labeled mouse anti-human eosinophil surface marker CD49d-IgG or rat anti-mouse eosinophil surface marker Siglec-F-IgG (BD Biosciences) [22,23]. The Hu- or Ms-monocytes were pre-treated with 50 ng/mL M-CSF (Sigma) at 37°C for 5 d to differentiate them into macrophages (Hu- or Ms-macrophages) before use and the differentiated macrophages were identified by flow cytometry using FITC-labeled mouse anti-human macrophage surface marker CD163-IgG or rat anti-mouse macrophage surface marker F4/80-IgG (BD Biosciences) [24].

### Isolation of primary human or mouse neutrophils

Human or murine peripheral blood neutrophils (Hu- or Ms-neutrophils) were isolated from healthy volunteers or from C3H/HeJ mice using human or mouse MicroBead neutrophil separation kit (Miltenyi Biotec) according to the manufacturer’s protocol. Briefly, EDTA-anticoagulated human or mouse peripheral blood samples were mixed with lysis buffer to lyse erythrocytes for 10 min and then centrifuged at 300×g for 10 min at room temperature. The cell pellets were suspended in 10 mM phosphate buffered saline (PBS, pH7.4) and then filtered with a 40 μm cell strainer. The collected cells were washed twice with PBS and then incubated in human CD66abce-biotin or mouse neutrophil biotin cocktail at 4°C for 10 min. The cell suspensions were mixed with Anti-Biotin magnetic beads for a 15-min incubation at 4°C and then the neutrophils were isolated by LS magnetic separation column. The purity (>95%) of isolated Hu- or Ms-primary neutrophils was detected by flow cytometry as above using human neutrophil surface maker CD15-APC or mouse neutrophil surface maker Ly6G-APC (eBioscience). In addition, the possible contamination of eosinophils and monocytes in the isolated Hu- or Ms-neutrophils was also tested by flow cytometry using FITC-labeled mouse anti-human eosinophil surface marker CD49d-IgG or BB515-labeled rat anti-mouse eosinophil surface marker Siglec-F-IgG (BD Biosciences) and FITC-labeled mouse anti-human monocyte surface marker CD14-IgG or rat anti-mouse monocyte surface marker CD11b-IgG (BD Biosciences) [25,26].

### Detection of intracellular leptospires after phagocytosis

Hu- and Ms-macrophages and Hu- or Ms-neutrophils (10^6^ per well) were respectively seeded in 6-well culture plates (Corning, USA) for incubation overnight at 37°C to form cell monolayers. Freshly cultured *L*. *interrogans* strain Lai was collected by centrifugation at 13,800×g for 15 min at 15°C and then washed twice with PBS. The leptospiral pellet was suspended in antibiotic-free 2.5% FCS RPMI-1640 medium for counting with a Petroff-Hausser counting chamber (Fisher Scientific, USA) under a dark-field microscope [27]. The cell monolayers were thoroughly washed with PBS and then infected with the spirochetes (1×10^8^) at a multiplicity of infection (MOI) of 100 (100 leptospires per cell) for 1, 2, 4, 8, 12 or 24 h [27,28]. After trypsinization and washing thoroughly with PBS, the co-cultures were centrifuged at 400×g for 10 min (4°C) to precipitate the extracellular leptospire-free cells and the harvested cells were counted using a Type Cedex XS automatic cell counter (Innovatis, USA). The cells were fixed with 4% paraformaldehyde-PBS for 30 min, and then permeabilized with 0.1% Triton X100-PBS for 30 min to allow antibodies to penetrate into the cells. After blocking with 5% BSA-PBS and washing with PBS, the cells were incubated with 1:100 diluted rabbit anti-*L*. *interrogans* strain Lai-IgG made in our laboratory [29], and 1:500 diluted Alexa Fluor 594-conjugated goat anti-rabbit-IgG (Invitrogen, USA) for 1 h at room temperature, respectively, to stain intracellular leptospires. After washing with PBS again, the cells were incubated with 1 μg/mL DAPI (Invitrogen) for 15 min to stain the cell nucleus. Finally, the cells were smeared on glass slides, and the intracellular leptospires (red) around the cell nucleus (blue) were observed under a laser confocal microscope (Olympus FV1000, Japan) (590 nm excitation and 617 nm emission wavelengths for Alexa Fluor594 detection, and 355 nm excitation and 460 nm emission wavelengths for DAPI detection). In addition, the *Leptospira*-infected cells were fixed, dehydrated, embedded, sectioned and stained as previously described [30], and the intracellular leptospires in phagosomes were observed under a transmission electron microscope (Philips TECNAI-10, Holland). Normal cells without infection were used as the controls.

### Enumeration of intracellular leptospires

The extracellular leptospire-free *L*. *interrogans* strain Lai-infected Hu- or Ms-macrophages and Hu- or Ms-neutrophils obtained as described above were lysed with 0.05% sodium deoxycholate-PBS (Sigma), followed by a short centrifugation at 500×g to remove cell debris. The supernatants were collected by another centrifugation at 13,800×g for 15 min (4°C) to precipitate intracellular leptospires. The leptospiral pellets were suspended in PBS for counting under a dark-field microscope with a Petroff-Hausser counting chamber (Fisher Scientific) as described above.

### Detection of living or death of intracellular leptospires

The monolayers of Hu- or Ms-macrophages and Hu- and Ms-neutrophils were prepared and infected with *L*. *interrogans* strain Lai as described above. After trypsinization and washing thoroughly with PBS, the co-cultures were centrifuged at 400×g for 10 min (4°C) to precipitate the extracellular leptospire-free cells. The collected cells were lysed with 0.05% sodium deoxycholate-PBS, followed by a short centrifugation at 500×g to remove cell debris. The supernatants were centrifuged at 13,800×g for 15 min (15°C) to precipitate intracellular leptospires. Subsequently, the living or dead leptospires were visualized using a LIVE/DEAD BacLight bacterial viability kit (Molecular Probes, USA) as previously described [31]. Briefly, the leptospires from infected cells were stained with two different fluorescent nucleic dyes, SYTO^®^ 9 and propidium iodide (PI), for a 15-min incubation at room temperature, and then detected using a laser confocal microscope (Olympus FV1000, Japan) and a spectrofluorimeter (Molecular Devices, USA) (485 nm excitation and 630 nm emission wavelength for SYTO^®^ 9 detection, and 485 nm excitation and 530 nm emission wavelength for PI detection). The confocal microscopic data contained the images of SYTO^®^ 9-stained living (green) or PI-stained dead (red) leptospires and the fold changes of red fluorescence intensity were used for semi-quantification of the dead leptospires. In addition, the percentages of living or dead intracellular leptospires in the infected macrophages and neutrophils were determined by spectrofluorimetry. In parallel, the leptospires (10^6^/mL) from infected cells were serially diluted with EMJH-liquid medium and incubated onto EMJH-agar plates for 3 weeks at 28°C. The leptospiral colony-forming units (CFUs) were counted after incubation. In addition, the leptospires (10^6^/mL) from infected cells were inoculated into EMJH-liquid medium for a 7-d incubation at 28°C, and then counted under a dark-field microscope with a Petroff-Hausser counting chamber (Fisher Scientific) to investigate the difference of their growth and proliferation [32]. In the detections, the same number of spirochetes from culture in EMJH-liquid medium was used as the control.

### Detection of fusion of leptospiral phagosomes with lysosomes

The monolayers of Hu- or Ms-macrophages and Hu- or Ms-neutrophils were prepared and then infected with *L*. *interrogans* strain Lai as described above. After trypsinization and washing thoroughly with PBS, the co-cultures were centrifuged at 400×g for 10 min (4°C) to precipitate the cells. The subsequent washing, fixation, permeabilization and blockage of the collected cells were the same as described above. The cells were incubated with 1:100 diluted rabbit anti-*L*. *interrogans* strain Lai-IgG made in our laboratory [29], rat anti-human or mouse LAMP-1-IgG (Abcam, UK) for overnight at 4°C. After washing with PBS, the cells were stained with 1:500 diluted Alexa Fluor594-conjugated goat anti-rabbit-IgG (Invitrogen) or Alexa Fluor488-conjugated goat anti-rat-IgG (Abcam) for 1 h at room temperature. After washing with PBS again, the cells were stained with DAPI as described above. Finally, the cells were smeared on glass slides and then observed under a laser confocal microscope (Olympus FV1000, Japan) (the excitation and emission wavelengths for Alexa Fluor594 or DAPI detection as described above while 495 nm excitation and 519 nm emission wavelengths for Alexa Fluor488 detection). Co-localization (yellow) of the intracellular leptospires (red) and LAMP-1 (green), a lysosomal marker [33], indicates the fusion of the leptospiral phagosomes with lysosomes. The percentages of leptospires co-localizing with lysosomes were analyzed using Metamorph 7.7.6 software (UIC, USA).

### Detection of total ROS levels in *Leptospira*-infected cells

The monolayers of Hu- or Ms-macrophages and Hu- or Ms-neutrophils were prepared and infected with *L*. *interrogans* strain Lai as described above. After trypsinization and washing thoroughly with PBS, the co-cultures were centrifuged at 400×g for 10 min (4°C) to precipitate the cells. The cell pellets were suspended in antibiotic-free 2.5% FCS RPMI-1640 medium containing 10 μM dichlorofluorescein diacetate (DCFH-DA) (Sigma), a ROS-specific fluorescent dye, for a 30-min incubation at 37°C [21]. After washing with PBS again, the total intracellular ROS was detected by laser confocal microscopy (488 nm excitation and 530 nm emission wavelengths) and the fluorescence intensity was measured to reflect the total intracellular ROS levels. Normal cells without infection were used as the controls.

### ROS inhibition test

In order to determine the influence of ROS levels on the viability of intracellular leptospires after phagocytosis, all the four tested phagocytes were pretreated with 10 mM N-acetyl-L-cysteine (NAC) (Sigma, USA), a ROS scavenger, for 1 h at 37°C [34]. The subsequent experimental steps, such as infection with *L*. *interrogans* strain Lai, isolation of intracellular leptospires, and detection of living or dead intracellular leptospires by spectrofluorimetry were the same as described above. In the test, NAC-untreated but *L*. *interrogans* strain Lai-infected macrophages and neutrophils were used as the controls.

### Detection of NO levels in *Leptospira*-infected cells

The monolayers of Hu- or Ms-macrophages and Hu- or Ms-neutrophils were prepared and infected with *L*. *interrogans* strain Lai as described above. After trypsinization and washing thoroughly with PBS, the co-cultures were centrifuged at 400×g for 10 min (4°C) to precipitate the cells. The cell pellets were suspended in antibiotic-free 2.5% FCS RPMI-1640 medium containing 5 μM 4-amino-5-methylamino-2’,7’-difluorescein diacetate (DAF-FM DA) (Sigma), a NO-specific fluorescent probe, for a 20-min incubation at 37°C [35]. After washing with PBS again, the intracellular NO was detected by laser confocal microscopy (495 nm excitation and 515 nm emission wavelengths) and the fluorescence intensity was measured to reflect NO levels. Normal cells without infection were used as the controls.

### NO-blockage test

To determine the influence of NO levels on the viability of intracellular leptospires after phagocytosis, all the four tested phagocytes were pretreated with 100 μM S-methylisothiourea (SMT) (Sigma), an inhibitor of inducible nitric oxide synthase (iNOS) [36], for 30 min at 37°C. The subsequent experimental steps, such as infection with *L*. *interrogans* strain Lai, isolation of intracellular leptospires, and detection of living or dead leptospiral percentages by spectrofluorimetry were the same as described above. In the test, SMT-untreated but *L*. *interrogans* strain Lai-infected macrophages and neutrophils were used as the controls.

### Detection of intracellular free Ca^2+^ concentrations during infection

The monolayers of Hu- or Ms-macrophages and Hu- or Ms-neutrophils were prepared and then infected with *L*. *interrogans* strain Lai as described above. After trypsinization and washing thoroughly with PBS, the co-cultures were centrifuged at 400×g for 10 min (4°C) to precipitate the cells. The cell pellets were suspended in antibiotic-free 2.5% FCS RPMI-1640 medium containing 10 μM fluo-4/AM (Invitrogen), a fluorescent probe of intracellular free Ca^2+^, for a 30-min incubation at 37°C [37]. After washing with PBS again, the intracellular free Ca^2+^ was detected by laser confocal microscopy (494 nm excitation and 516 nm emission wavelengths) and the fluorescence intensity was measured to reflect intracellular free Ca^2+^ concentrations ([Ca^2+^]i). Normal cells without infection were used as the controls.

### Intracellular free Ca^2+^ chelation test

To determine the influence of [Ca^2+^]i on the viability of intracellular leptospires after phagocytosis, all the four tested phagocytes were pretreated with 100 μM 1,2-bis (2-aminophenoxy) ethane-N, N, N’, N’-tetraacetic acid/AM (BAPTA/AM) (Sigma), an intracellular free Ca^2+^ chelator [37]. The subsequent experimental steps, such as infection with *L*. *interrogans* strain Lai, isolation of intracellular leptospires, and detection of living or dead leptospires by spectrofluorimetry, were the same as described above. In the test, BAPTA/AM-untreated but *L*. *interrogans* strain Lai-infected macrophages and neutrophils were used as the controls.

### Generation of *Leptospira*-infected mice

Previous reports showed that C3H/HeJ mice could be used to generate *Leptospira*-infected mouse model [27,38–40]. In this study, the mice were intraperitoneally injected with 0.25 mL culture containing 1×10^7^ *L*. *interrogans* strain Lai, and five animals were used per group. Five negative control animals were intraperitoneally injected with the same volume of EMJH liquid medium. The animals were monitored twice daily. According to the general course of leptospirosis, lung, liver and kidney samples from the infected animals were collected on days 3, 5 and 7 after challenge were collected for histological examination after HE-staining [32]. Moreover, the leptospires in the three types of tissues were observed under an optical microscope after silver staining [32].

### Detection of infiltrated phagocytes in tissues of *Leptospira*-infected mice

The lung, liver and kidney tissue samples from *Leptospira*-infected mice mentioned above were fixed with 4% formalin and then embedded and sectioned. CD11b or Ly6G has been considered as mouse peripheral blood-derived mononuclear-macrophage or neutrophil surface makers, respectively [41,42]. Using 1:100 diluted rabbit anti-mouse CD11b or rat anti-mouse Ly6G-IgG as the primary antibody (Abcam) and 1:1000 diluted HRP-conjugated goat anti-rabbit-IgG or goat anti-rat-IgG as the secondary antibody (Abcam), an immunohistochemical method was used to detect the mononuclear-macrophages and neutrophils in the tissues from peripheral blood. The stained mononuclear-macrophages and neutrophils (brown) per mm^2^ section were quantitatively estimated using Image-Pro Plus software (Nikon, Japan) [43–45]. In the detection, the mice injected with EMJH liquid medium were used as the control. In addition, the efficiencies of the CD11b-IgG and Ly6G-IgG were determined using M-CSF-induced Ms-macrophages and Ms-neutrophils from peripheral blood before use.

### Detection of chemokines in sera of *Leptospira*-infected mice and leptospirosis patients

Chemokines in the serum samples from five *Leptospira*-infected mice that collected above and from five leptospirosis patients were detected using quantitative mouse chemokine antibody microarray-Q1, and human chemokine antibody microarray-AAH-CHE-G1 (RayBiotech, USA) as previously described [27,45]. Sera from five leptospirosis patients (males, aged 25, 28, 29, 31 and 35 years) were provided by the Center for Disease Prevention and Control of Zhejiang Province, China. The patients had a typical clinical manifestations of leptospirosis, confirmed by the presence of visible leptospires in peripheral blood specimens by dark-field microscopy and subsequent fractional cultivation [12]. The green fluorescence signals were captured and analyzed using an InnoScan 300 Microarray laser scanner. In the detections, the serum samples from five mice injected with EMJH liquid medium and from five healthy individuals were used as the controls.

### Detection of VECAMs in tissues of *Leptospira*-infected mice

The lung, liver and kidney tissue samples from *Leptospira*-infected mice mentioned above were fixed with 4% formalin and then embedded and sectioned. Using 1:100 diluted rabbit anti-mouse VCAM-1, ICAM-1, E- or P-selectin-IgG (Abcam) as the primary antibody and 1:1000 diluted HRP-conjugated goat anti-rabbit-IgG (Abcam) as the secondary antibody, an immunohistochemical method was used to detect these factors in the tissue samples and the expression of the VECAMs were quantitatively estimated using Image-Pro Plus software as described above. In the detection, the mice injected with EMJH liquid medium were used as the controls.

### Statistical analysis

Data from a minimum of at least three experiments were averaged and presented as mean ± standard deviation (SD). One-way analysis of variance (ANOVA) followed by Dunnett’s multiple comparisons test were used to determine significant differences. Statistical significance was defined as *p*<0.05.

## Results

### Macrophages act as the major phagocytes against *L*. *interrogans*

**Fig 1:**
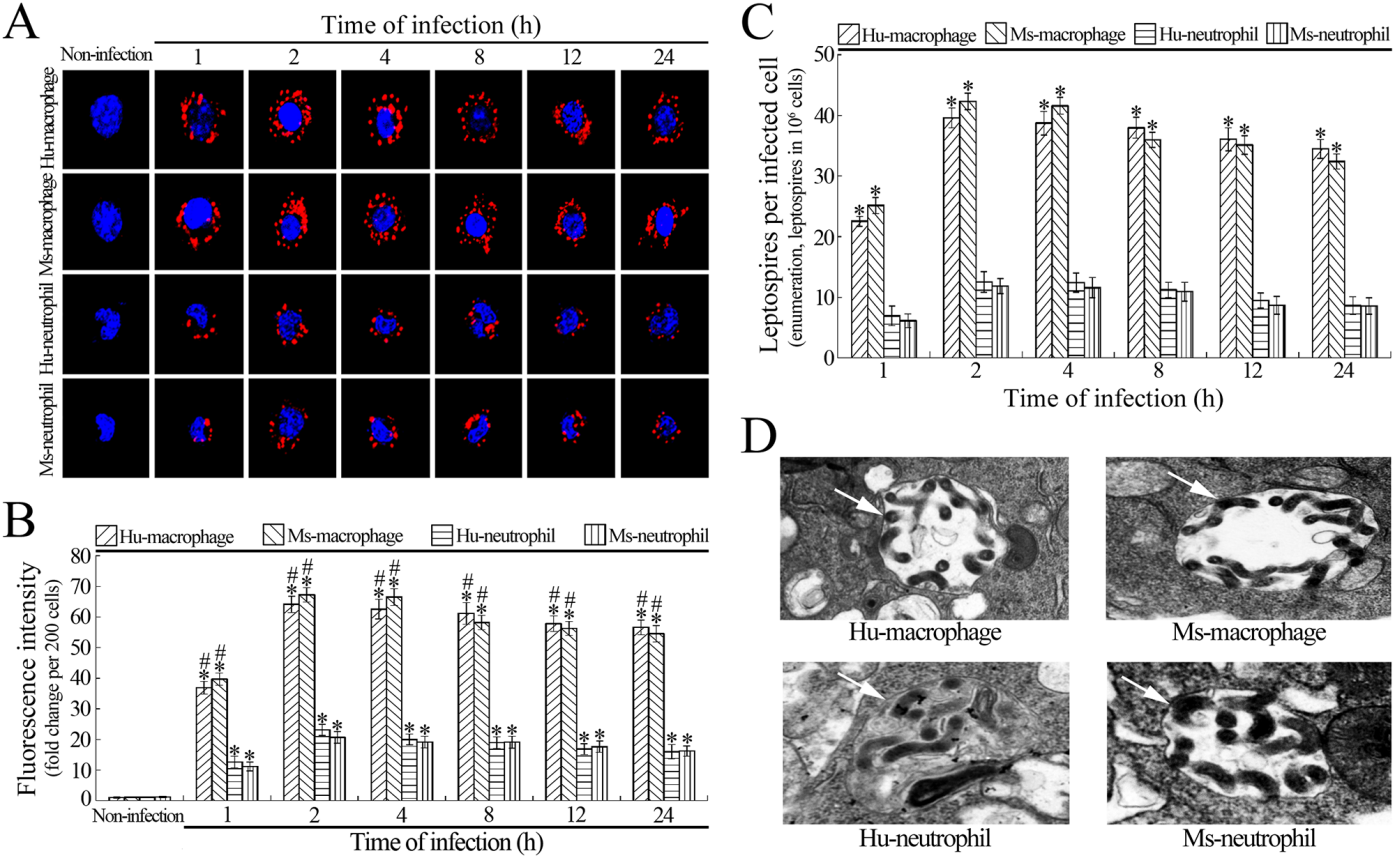
Macrophages are the main phagocytes to phagocytose leptospires. **(A)** Leptospiral phagocytosis in *L*. *interrogans*-infected macrophages and neutrophils, determined by confocal microscopy for the indicated infection times. The blue plaques indicate the nucleus. The red spots around the nucleus indicate the intracellular leptospires. **(B)** Statistical summary of red fluorescence intensity reflecting the intracellular leptospires in *L*. *interrogans*-infected macrophages and neutrophils. Statistical data from experiments such as shown in A. Bars show the means ± SD of three independent experiments. Two hundred cells in each experiment were analyzed to quantify the intensities of red fluorescence. The means of red fluorescence intensities from the cells without infection were set as 1.0. *: *p*<0.05 *vs* the red fluorescence intensities from the cells without infection. ^#^: *p*<0.05 *vs* the red fluorescence intensities from the neutrophils for the indicated times during infection. **(C)** Leptospiral numbers in *L*. *interrogans*-infected macrophages and neutrophils, determined by leptospiral enumeration for the indicated infection times. Bars show the means ± SD of three independent experiments. 10^6^ cells per experiment were used for leptospiral counting. *: *p*<0.05 *vs* the leptospiral numbers in the neutrophils for the indicated times during infection. **(D)** Leptospiral phagosomes in *L*. *interrogans* strain Lai-infected macrophages and neutrophils, detected by the transmission electron microscopy. The arrows indicate the intracellular leptospiral phagosomes.

The purity of isolated Hu- or Ms-monocytes was 97.4% or 97.7% while the purity of isolated Hu- or Ms-neutrophils was 98.9% or 98.5% (shown in S1 File). The ratios of contaminated eosinophils and neutrophils in the isolated Hu- or Ms-monocytes and of contaminated eosinophils and monocytes in the isolated Hu- or Ms-neutrophils were less than 0.2% and 0.4% (shown in S1 File), respectively. After induction with M-CSF, 85.7% of the Hu-monocytes and 87.4% of the Ms-monocytes were differentiated into macrophages (shown in S1 File). The confocal microscopic examination confirmed that the number of leptospires in the *L*. *interrogans*-infected Hu- or Ms-macrophages was significantly higher than in the *L*. *interrogans*-infected Hu- or Ms-neutrophils (Fig 1A and 1B). The intracellular leptospiral enumeration also confirmed that the macrophages phagocytosed more leptospires than the neutrophils (Fig 1C). The electron microscopic examination showed that the intracellular leptospires had a curled shape, surrounded by a membrane (Fig 1D). The data suggested that macrophages have a higher *L*. *interrogans*-phagocytosing ability than neutrophils.

### Macrophages but not neutrophils are the main phagocytes killing *L*. *interrogans*

**Fig 2:**
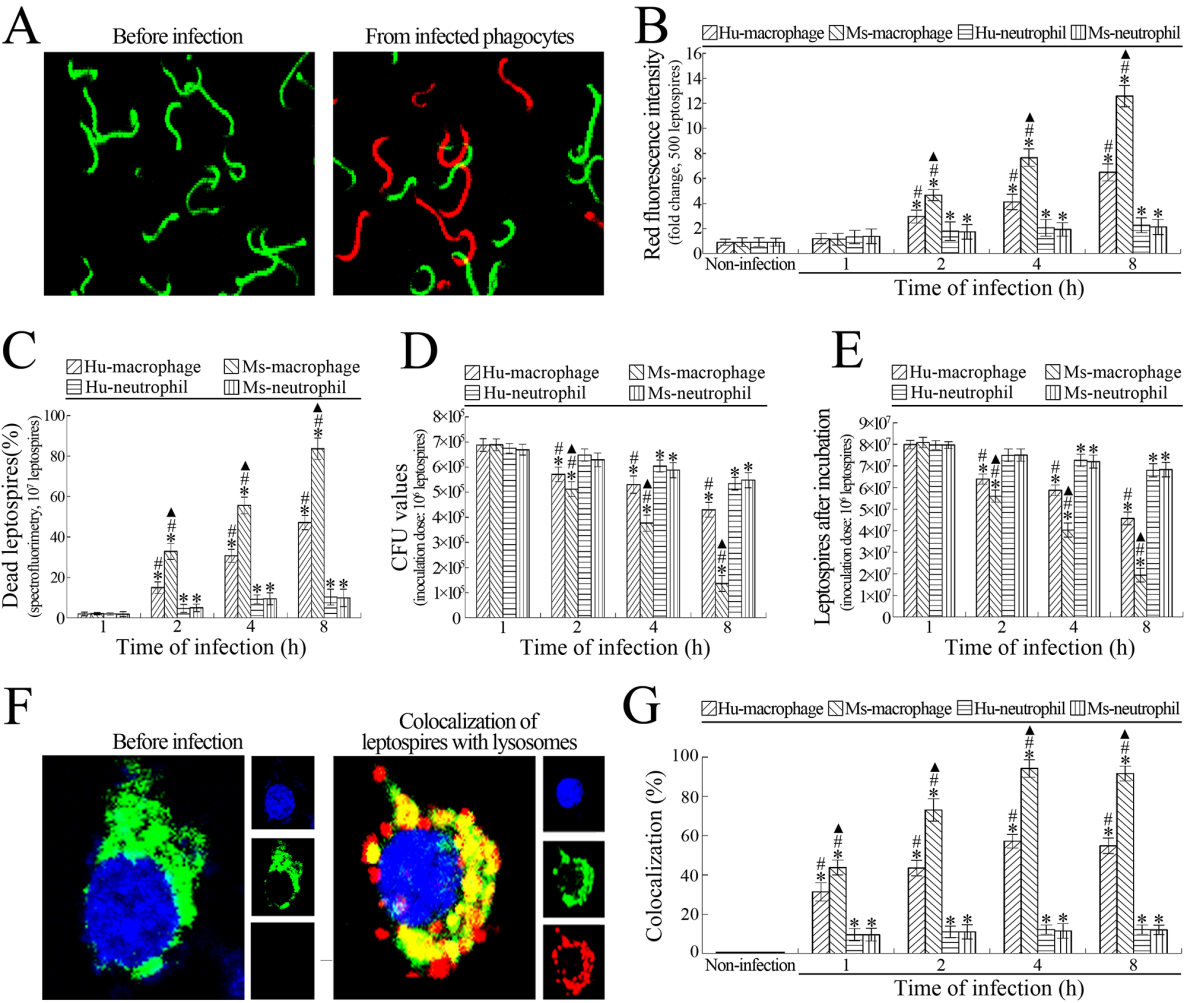
Macrophages have a higher ability of killing leptospires than neutrophils. **(A)** Living and dead leptospires under a confocal microscope. The green leptospires were living while the red leptospires were dead. **(B)** Ability of macrophages and neutrophils to kill intracellular leptospires during infection with *L*. *interrogans*, determined by confocal microscopy for the indicated infection times. Bars show the means ± SD of three independent experiments. Five hundred leptospires in each experiment were analyzed to quantify the values of red fluorescence intensity. The means of red fluorescence intensities from the leptosires without infection were set as 1.0. *: *p*<0.05 *vs* the red fluorescence intensities of leptospires without infection. ^#^: *p*<0.05 *vs* the red fluorescence intensities reflecting the dead leptospires from the infected Hu- or Ms-neutrophils. ^▲^: *p*<0.05 *vs* the red fluorescence intensities reflecting the dead leptospires from the infected Hu-macrophages. **(C)** Percentages of dead leptospires from macrophages and neutrophils during infection with *L*. *interrogans*, determined by spectrofluorimetry for the indicated infection times. Bars show the means ± SD of three independent experiments. 10^7^ leptospires in each experiment were used to determine the dead leptospiral percentages. *: *p*<0.05 *vs* the dead percentages of the leptospires without infection. ^#^: *p*<0.05 *vs* the dead leptospiral percentages from the infected Hu- or Ms-neutrophils. ^▲^: *p*<0.05 *vs* the dead leptospiral percentages from the infected Hu-macrophages. **(D)** Fewer leptospiral colonies from *L*. *interrogans*-infected macrophages than neutrophils, assessed by CFU enumeration for the indicated infection times. Bars show the means ± SD of three independent experiments. 10^6^ leptospires from each of the infected cells were serially diluted and then inoculated onto EMJH-agar plates for a three-week incubation at 28°C for CFU enumeration. *: *p*<0.05 *vs* the CFU values of the leptospires without infection. ^#^: *p*<0.05 *vs* the CFU values of the leptospires from the infected Hu- or Ms-neutrophils. ^▲^: *p*<0.05 *vs* the CFU values of the leptospires from the infected Hu-macrophages. **(E)** Attenuated growth ability of leptospires from *L*. *interrogans*-infected macrophages than neutrophils, assessed by leptospiral enumeration after incubation. Bars show the means ± SD of three independent experiments. 10^6^ leptospires from each of the infected cells were inoculated in EMJH liquid medium for one-week incubation at 28°C for leptospiral enumeration. *: *p*<0.05 *vs* the growth ability of the leptospires without infection. ^#^: *p*<0.05 *vs* the growth ability of the leptospires from the infected Hu- or Ms-neutrophils. ^▲^: *p*<0.05 *vs* the growth ability of the leptospires from the infected Hu-macrophages. **(F)** Co-localization of intracellular leptospires with lysosomes under a confocal microscope. Three fluorescence images were merged in the left panels and separate fluorescence channels in the right panel. The blue plaques indicate the nucleus. The green plaques around the nucleus indicate the lysosomes. The red spots around the nucleus indicate the intracellular leptospires. The yellow spots or plaques indicate the co-localization of intracellular leptospires with lysosomes. **(G)** Co-localization of intracellular leptospires with lysosomes in *L*. *interrogans*-infected macrophages and neutrophils, determined by confocal microscopy for the indicated infection times. Bars show the means ± SD of three independent experiments. One hundred cells in each experiment were analyzed to quantify the intensities of yellow fluorescence. The means of yellow fluorescence intensities from the cells without infection were set as 1.0. *: *p*<0.05 *vs* the yellow fluorescence intensities of the leptospires without infection. ^#^: *p*<0.05 *vs* the yellow fluorescence intensity reflecting the intracellular leptospire-lysosome co-localization in the infected Hu- or Ms-neutrophils. ^▲^: *p*<0.05 *vs* the yellow fluorescence intensity reflecting the intracellular leptospire-lysosome co-localization in the infected Hu-macrophages.

The confocal microscopic and spectrofluorometric examinations confirmed that the numbers and percentages of dead leptospires in *L*. *interrogans*-infected Hu- or Ms-macrophages were continuously increased from 2 to 8 h postinfection during a 24-h infection (Fig 2A–2C). The results of enumeration demonstrated that the CFU and growth ability of the leptospires from both infected Hu- and Ms- macrophages were continuously decreased from 2 to 8 h postinfection (Fig 2D and 2E). However, the dead leptospiral numbers and percentages as well as the leptospiral CFU values and growth ability from the infected macrophages remained constant from 8 to 24 h postinfection (data not shown). Compared to the data from the infected macrophages above, the numbers and percentages of dead leptospires in the *L*. *interrogans*-infected Hu- or Ms-neutrophils only displayed a mild increase at 2 h after infection (Fig 2B and 2C) while the leptospiral CFU and growth ability displayed a slight decrease from 4 to 8 h postinfection (Fig 2D and 2E). However, the dead leptospiral numbers and percentages as well as the leptospiral CFU and growth ability from the infected neutrophils in the subsequent infection process showed no significant change (data not shown). Besides, the Ms-macrophages displayed a higher ability to kill the phagocytized leptospires than Hu-macrophages (Fig 2B–2E). The data suggested that macrophages rather than neutrophils act as the main phagocytes that eliminates leptospires in infected animals and leptospirosis patients.

### Higher fusion of phagosomes harboring leptospires with lysosomes in macrophages than neutrophils

The confocal microscopic examination showed that most of the intracellular leptospires in the *L*. *interrogans*-infected Ms-macrophages were co-localized with LAMP-1, a lysosomal marker [33], but approximately half of the intracellular leptospires in the infected Hu-macrophages and a few of the intracellular leptospires in the infected Hu- or Ms-neutrophils co-localized with LAMP-1 during a 24-h infection (Fig 2F and 2G). The co-localization levels of intracellular leptospires and lysosomes in both infected Hu- and Ms- macrophages were continuously increased from 1 to 4 h post-infection (Fig 2G), but the co-localization levels at from 4 to 24 h post-infection showed no significant change (data not shown). However, the co-localization levels of intracellular leptospires and lysosomes in the infected Hu- or Ms-neutrophils exhibited a mild increase at 1 h postinfection and then maintained the similar increases at the subsequent infection process (Fig 2G). The results suggested that macrophages, but not neutrophils, are the major phagocytes that kill *L*. *interrogans*, and mouse macrophages have a higher ability to kill *L*. *interrogans* than human macrophages.

### Macrophages use ROS to kill intracellular leptospires

**Fig 3:**
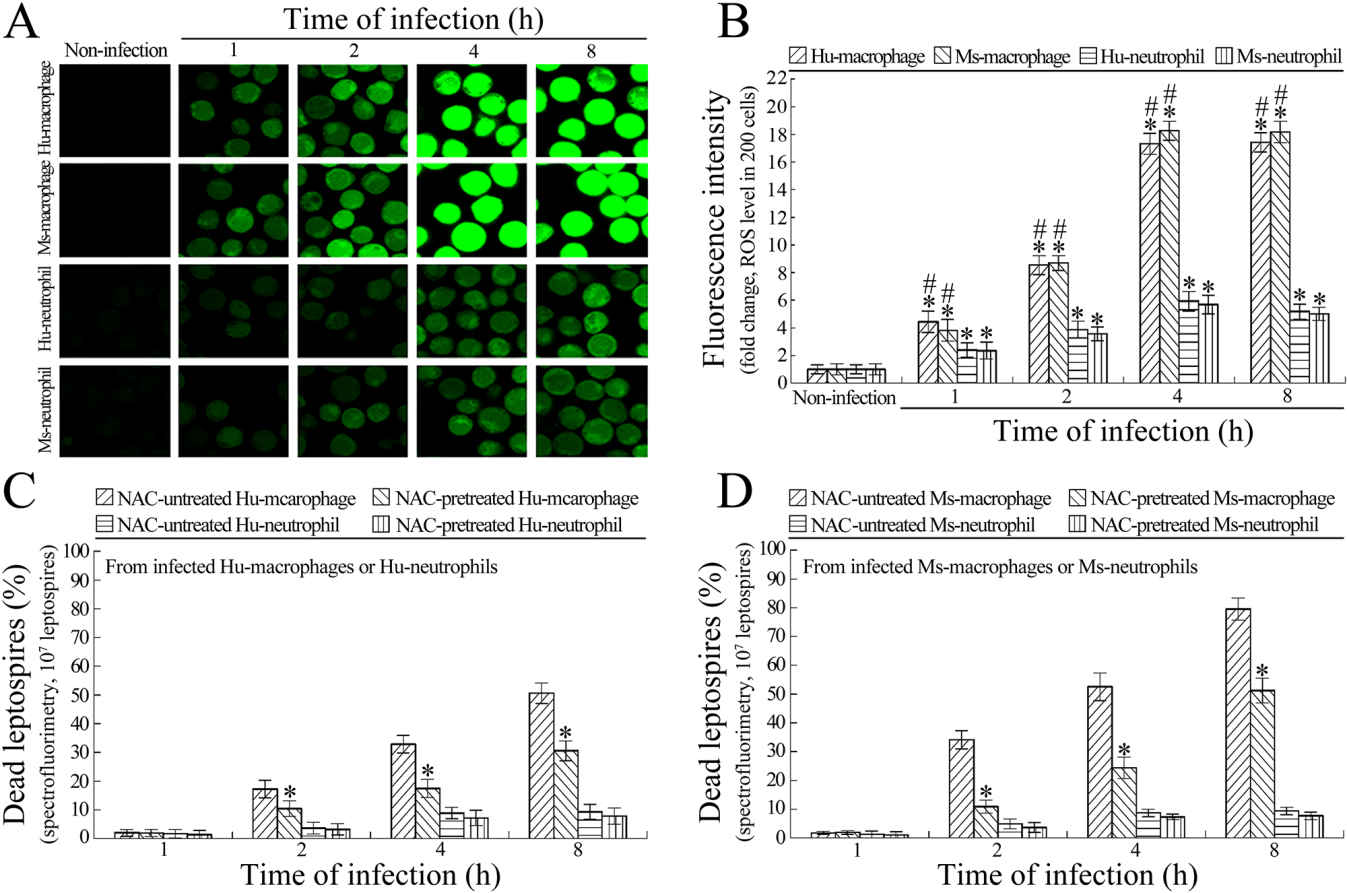
Higher ROS levels required by macrophages for killing intracellular leptospires. **(A)** Total ROS levels in *L*. *interrogans*-infected macrophages and neutrophils, determined by confocal microscopy for the indicated infection times. The green fluorescence indicates the intracellular total ROS levels. **(B)** Statistical summary of green fluorescence intensity reflecting the total ROS levels in *L*. *interrogans*-infected macrophages and neutrophils. Statistical data from experiments such as shown in A. Bars show the means ± SD of three independent experiments. Two hundred cells in each experiment were analyzed to quantify the values of fluorescence intensity. The means of fluorescence intensities from the cells without infection were set as 1.0. *: *p*<0.05 *vs* the total ROS level in the macrophages and neutrophils without infection. ^#^: *p*<0.05 *vs* the total ROS levels in the *L*. *interrogans*-infected neutrophils. **(C)** Lower dead leptospiral percentages in NAC-pretreated *L*. *interrogans*-infected Hu-macrophages rather than Hu-neutrophils, determined by spectrofluorimetry for the indicated infection times. Bars show the means ± SD of three independent experiments. 10^7^ leptospires in each experiment were used to determine the dead leptospiral percentages. *: *p*<0.05 *vs* the dead leptospiral percentages from the NAC-untreated *L*. *interrogans*-infected Hu-macrophages. **(D)** Lower dead leptospiral percentages in NAC-pretreated *L*. *interrogans*-infected Ms-macrophages rather than Ms-neutrophils, determined by spectrofluorimetry for the indicated infection times. The legend is the same as in C except that this experiment was for detection of Ms-macrophages and Ms-neutrophils.

The confocal microscopic examination showed that the total ROS levels in the *L*. *interrogans*-infected Hu- or Ms-macrophages were significantly higher than that in the infected Hu- or Ms-neutrophils (Fig 3A and 3B). When the macrophages and neutrophils were pretreated with NAC, a ROS scavenger [34], the spectrofluorometric examination revealed that the percentages of dead leptospires in both the Hu- and Ms-macrophages were significantly decreased compared with the NAC-untreated infected macrophages, but NAC pretreatment did not influence the percentages of dead leptospires in the infected Hu- and Ms-neutrophils (Fig 3C and 3D). The results suggested that macrophages require high total ROS levels for killing intracellular leptospires.

### NO contributes to killing of intracellular leptospires by macrophages

**Fig 4:**
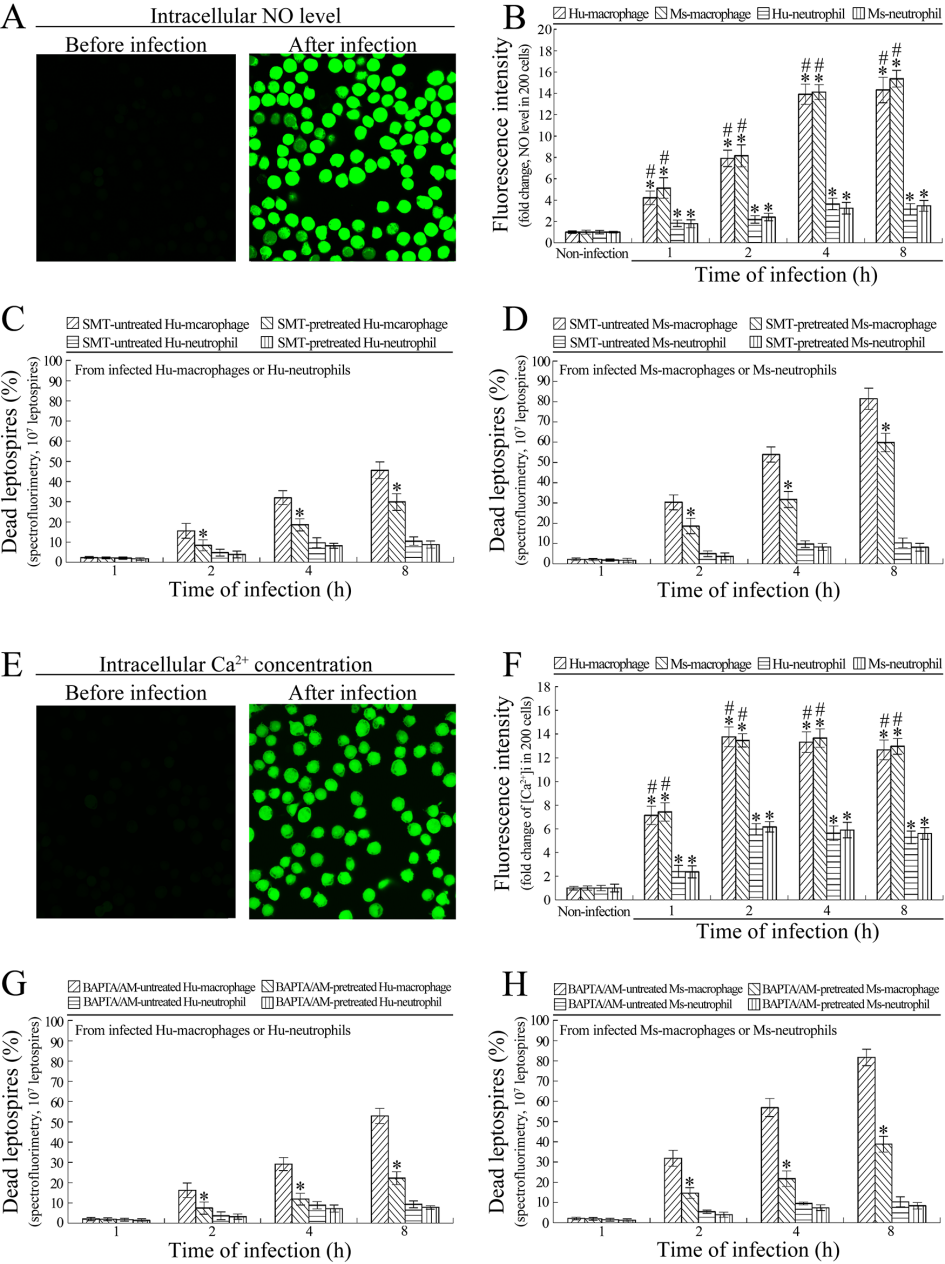
Higher NO levels and [Ca^2+^]i are necessary for macrophages to kill intracellular *L*. *interrogans*. **(A)** Intracellular NO in *L*. *interrogans*-infected macrophages or neutrophils under confocal microscope. The green fluorescence indicates the intracellular NO. **(B)** NO levels in *L*. *interrogans*-infected macrophages and neutrophils, determined by confocal microscopy for the indicated infection times. Bars show the means ± SD of three independent experiments. Two hundred cells in each experiment were analyzed to quantify the values of fluorescence intensity. The means of fluorescence intensities from the cells without infection were set as 1.0. *: *p*<0.05 *vs* the NO levels in the macrophages and neutrophils without infection. ^#^: *p*<0.05 *vs* the NO levels in the *L*. *interrogans*-infected neutrophils. **(C)** Lower dead leptospiral percentages in SMT-pretreated *L*. *interrogans*-infected Hu-macrophages rather than Hu-neutrophils, determined by spectrofluorimetry for the indicated infection times. Bars show the means ± SD of three independent experiments. 10^7^ leptospires in each experiment were used to determine the dead leptospiral percentages. *: *p*<0.05 *vs* the dead leptospiral percentages from the SMT-untreated *L*. *interrogans*-infected Hu-macrophages. **(D)** Lower dead leptospiral percentages in SMT-pretreated *L*. *interrogans*-infected Ms-macrophages rather than Ms-neutrophils, determined by spectrofluorimetry for the indicated infection times. The legend is the same as in C except that this experiment was for detection of Ms-macrophages and Ms-neutrophils. **(E)** Intracellular free Ca^2+^ in *L*. *interrogans*-infected macrophages or neutrophils under confocal microscope. The green fluorescence indicates the [Ca^2+^]i. **(F)** [Ca^2+^]i in *L*. *interrogans*-infected macrophages and neutrophils, determined by confocal microscopy for the indicated infection times. Bars show the means ± SD of three independent experiments. Two hundred cells in each experiment were analyzed to quantify the values of fluorescence intensity. The means of fluorescence intensities from the cells without infection were set as 1.0. *: *p*<0.05 *vs* the [Ca^2+^]i in the macrophages and neutrophils without infection. ^#^: *p*<0.05 *vs* the [Ca^2+^]i in the *L*. *interrogans*-infected neutrophils. **(G)** Lower dead leptospiral percentages in BAPTA/AM-pretreated *L*. *interrogans*-infected Hu-macrophages rather than Hu-neutrophils, determined by spectrofluorimetry for the indicated infection times. Bars show the means ± SD of three independent experiments. 10^7^ leptospires in each experiment were used to determine the dead leptospiral percentages. *: *p*<0.05 *vs* the dead leptospiral percentages from the BAPTA/AM-untreated *L*. *interrogans*-infected Hu-macrophages. **(H)** Lower dead leptospiral percentages in BAPTA/AM-pretreated *L*. *interrogans*-infected Ms-macrophages rather than Ms-neutrophils, determined by spectrofluorimetry for the indicated infection times. The legend is the same as in G except that this experiment was for detection of Ms-macrophages and Ms-neutrophils.

The confocal microscopic examination showed that the NO levels in the *L*. *interrogans*-infected Hu- or Ms-macrophages were significantly higher than in the infected Hu- or Ms-neutrophils (Fig 4A and 4B). When the macrophages were pretreated with SMT, an iNOS inhibitor [36], the spectrofluorometric examination revealed that the percentages of dead leptospires in the infected Hu- or Ms-macrophages were significantly decreased compared with the SMT-untreated infected macrophages, but SMT pretreatment did not influence the percentage of dead leptospires in the infected Hu- or Ms-neutrophils (Fig 4C and 4D). The results suggested that higher NO levels contribute to killing of intracellular leptospires by macrophages.

### [Ca^2+^]i is related to the ability of macrophages to kill intracellular leptospires

The confocal microscopic examination showed that the [Ca^2+^]i values in the *L*. *interrogans*-infected Hu- or Ms-macrophages were significantly higher than those in the infected Hu- or Ms-neutrophils (Fig 4E and 4F). When the macrophages were pretreated with BAPTA/AM, an intracellular free Ca^2+^ chelator [37], the spectrofluorometric examination revealed that the percentages of dead leptospires in the infected macrophages were significantly decreased compared with the BAPTA/AM-untreated infected Hu- or Ms-macrophages, but BAPTA/AM pretreatment did not influence the percentages of dead leptospires in the infected Hu- or Ms-neutrophils (Fig 4G and 4H). The data suggested that higher [Ca^2+^]i is closely related to the ability of macrophages to kill intracellular leptospires.

### Histopathological changes in *L*. *interrogans*-infected mice

**Fig 5:**
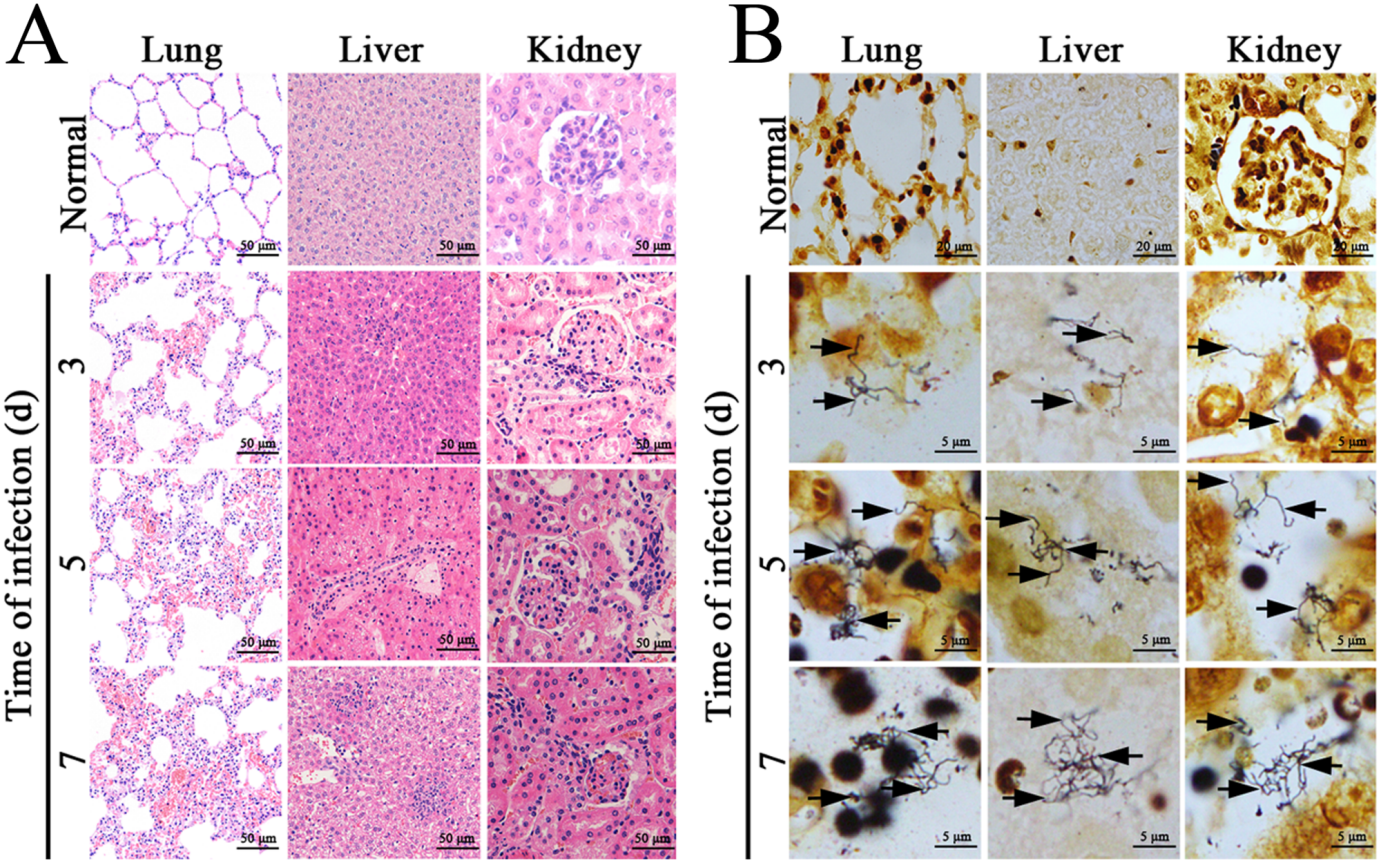
Histopathological changes and leptospires in tissues of *L*. *interrogans*-infected mice. **(A)** Histopathological changes in lung, liver and kidney tissues from *L*. *interrogans*-infected C3H/HeJ mice, examined by microscopy after HE staining. All the tissues had infiltration of inflammatory cells. The lung, liver and kidney tissues presented serious hemorrhage, extensive hepatocellular necrosis and serious congestion, respectively. **(B)** Visible leptospires in lung, liver and kidney tissues from *L*. *interrogans*-infected C3H/HeJ mice, examined by microscopy after silver staining. The arrows indicate leptospires in the three types of tissues from the infected animals.

The lung, liver and kidney tissue samples from *Leptospira*-infected C3H/HeJ mice presented typical histopathological changes of leptospirosis, such as inflammatory cell infiltration in the three types of tissues, hemorrhaging in lungs, extensive hepatocellular necrosis, and serious congestion in kidneys (Fig 5A). The silver-stained leptospires could be found in the lung, liver and kidney samples from the infected animals (Fig 5B).

### Mononuclear-macrophages are the main infiltrating phagocytes in tissues of *L*. *interrogans*-infected mice

**Fig 6:**
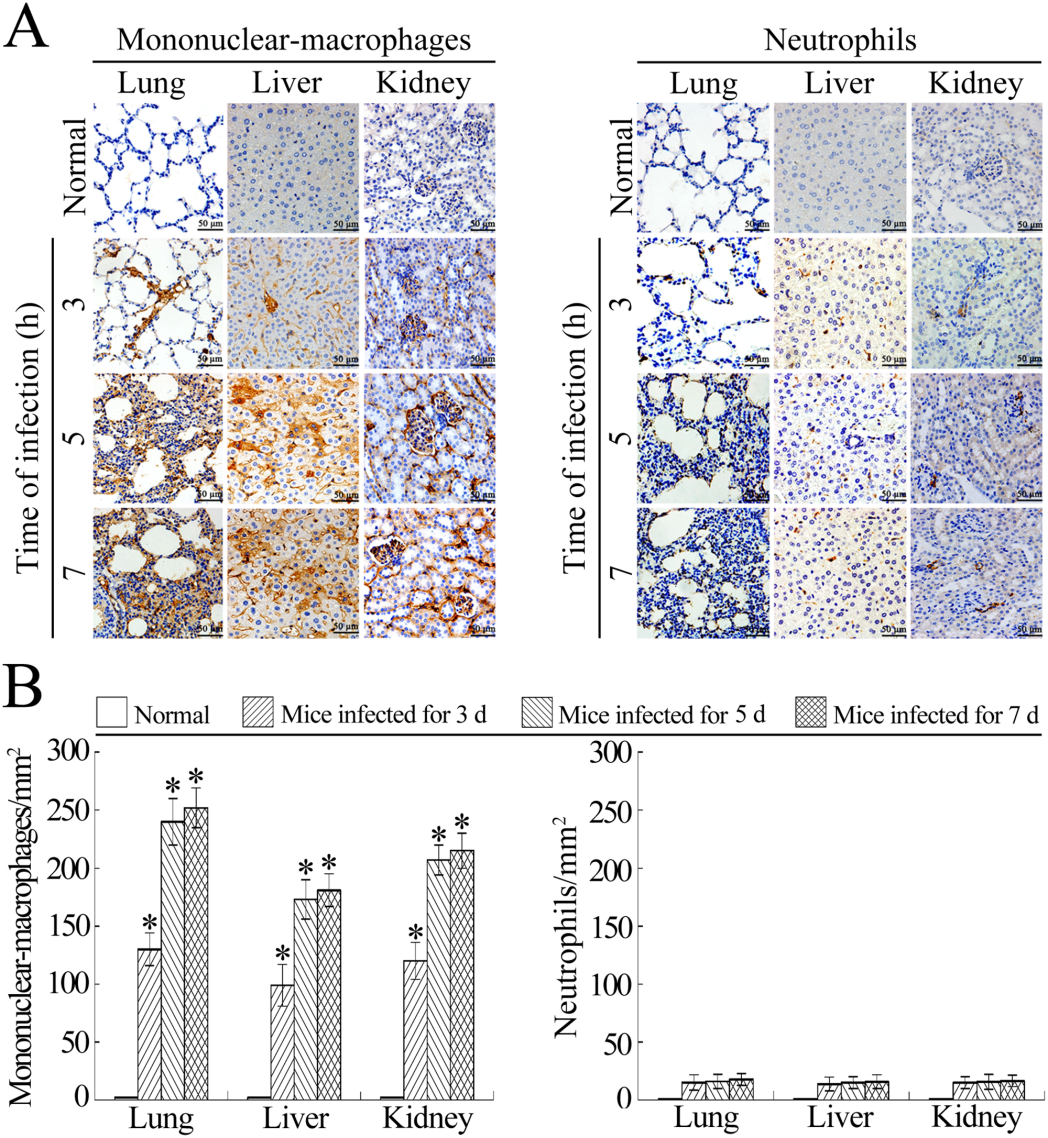
Mononuclear-macrophages from peripheral blood are the main infiltrating phagocytes during leptosirosis. **(A)** Infiltration of peripheral blood mononuclear-macrophages and neutrophils into the lung, liver and kidney tissues of *L*. *interrogans*-infected C3H/HeJ mice, visualized by immunohistochemistry for the indicated infection times. The mononuclear-macrophages or neutrophils were determined with CD11b or Ly6G antibody. **(B)** Infiltrated CD11b^+^ mononuclear-macrophages and Ly6G^+^ neutrophils in the lung, liver and kidney tissues from *L*. *interrogans*-infected mice, estimated by analysis using Image-Pro Plus software. Statistical data from experiments such as shown in A. Bars show the means ± SD of three independent experiments. *: *p*<0.05 *vs* the normal animals.

Histopathologically, peripheral blood monocyte-derived macrophages are called mononuclear-macrophages after they migrate into tissues [13,20]. Mice have been confirmed to possess a much lower proportion of neutrophils in peripheral blood leukocytes than humans [46]. However, neutrophils have been confirmed as the major infiltrating phagocytes in the lung and liver tissues of *Escherichia coli*- or *Streptococcus pneumoniae*-infected mice [47,48]. Although the antibodies used in this study could efficiently recognize both the mononuclear-macrophages and neutrophils from peripheral blood (S1 File and S1 Fig), the immunohistochemical examination showed that a large number of CD11b^+^ mononuclear-macrophages from peripheral blood were present in the lung, liver and kidney tissues of *L*. *interrogans*-infected mice, but few Ly6G^+^ neutrophils could be found in the tissues (Fig 6A and 6B). The results suggested that peripheral blood mononuclear-macrophages but not neutrophils are the major infiltrating phagocytes in *L*. *interrogans*-infected hosts.

### Increases in macrophage chemokine levels in sera of *Leptospira*-infected mice and leptospirosis patients

**Fig 7:**
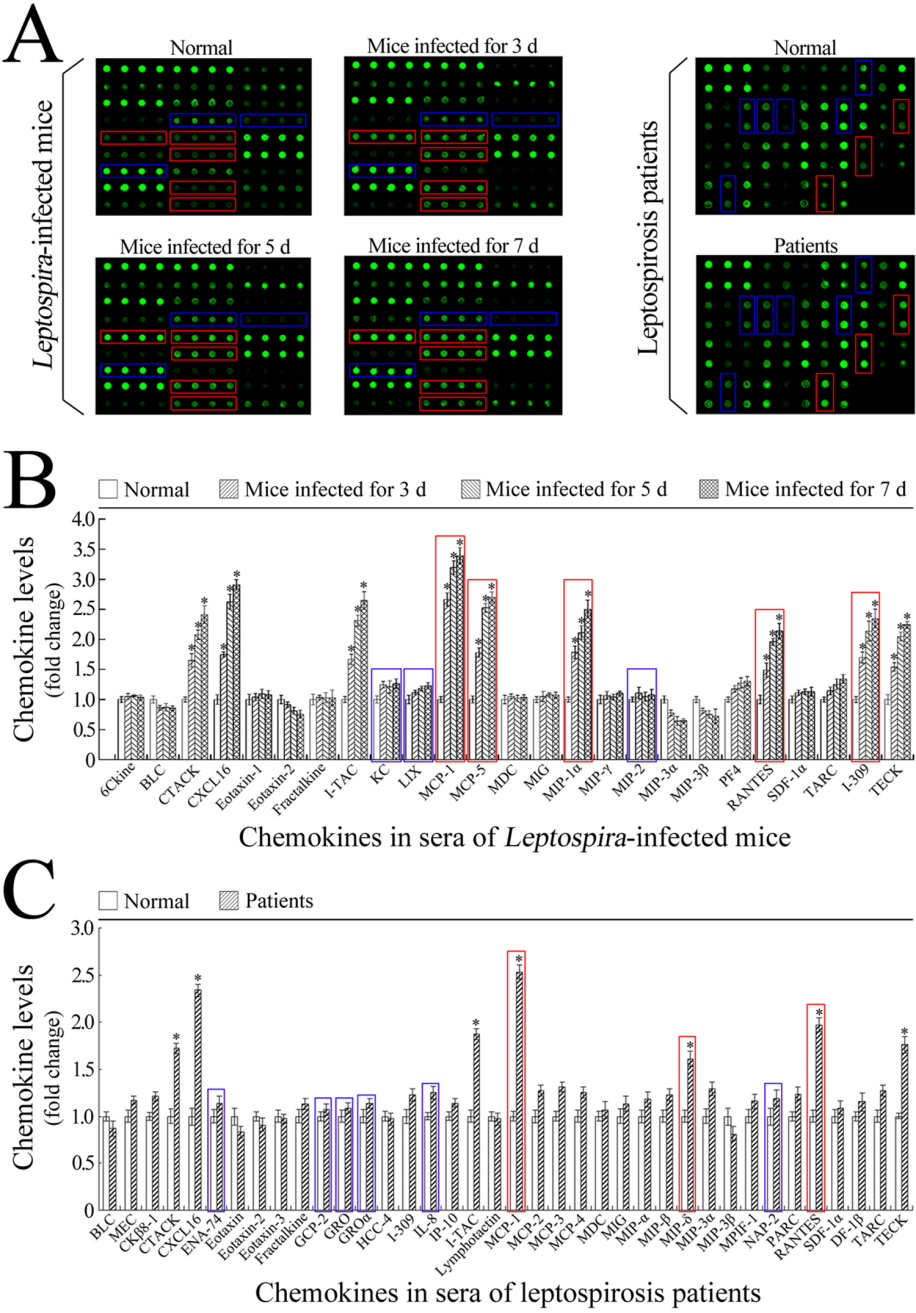
Profiles of chemokines of mononuclear-macrophages and neutrophils in serum samples from *L*. *interrogans*-infected mice and leptospirosis patients. **(A)** Detection results of the mouse and human chemokines of mononuclear-macrophages and neutrophils in sera from C3H/HeJ mice during infection with *L*. *interrogans* strain Lai, determined by chemokine detection microarrays for the indicated infection times. The spots within red frames indicate the mononuclear-macrophage chemokines, the spots within blue frames indicate the neutrophil chemokines. **(B)** Statistical summary of the chemokine profile in the serum samples from *L*. *interrogans*-infected mice. Statistical data from the microarray detection such as shown in A. Bars show the means ± SD of five independent serum samples. The means of chemokine levels in the serum samples from five mice without infection were set as 1.0. The bars within red frames indicate the mononuclear-macrophage chemokines and the bars within blue frames indicate the neutrophil chemokines. *: *p*<0.05 *vs* the chemokine levels in the serum samples from mice without infection. **(C)** Statistical summary of the chemokine profiles in the serum samples from leptospirosis patients. Statistical data from the microarray detection such as shown in A. Bars show the means ± SD of five patients. The means of chemokine levels in the serum samples from five healthy individuals were set as 1.0. The bars within red frames indicate the mononuclear-macrophage chemokines and the bars within blue frames indicate the neutrophil chemokines. *: *p*<0.05 *vs* the chemokine levels in the serum samples from five healthy individuals.

The mouse chemokine detection microarray confirmed that the levels of MCP-1, MCP-5, MIP-1α, RANTES and I-309, the macrophage chemokines, but not the levels of KC, LIX, and MIP-2, the neutrophil chemokines, in the sera of *L*. *interrogans*-infected C3H/HeJ mice were significantly increased during infection (Fig 7A and 7B). Similarly, the levels of macrophage chemokines such as MCP, MIP-δ and RANTES, but not the tested neutrophil chemokines (ENA-74, GCP-2, GRO, GROα, IL-8 and NAP-2) in the sera of leptospirosis patients were notably increased (Fig 7A and 7C). Besides, the levels of lymphocyte chemokines (CTACK and TECK) and natural killer (NK) cell chemokines (CXCL16 and I-TAC) also increased significantly (Fig 7B and 7C). The results suggested that the high levels of macrophage chemokines induce the infiltration of mononuclear-macrophages from peripheral blood into infected tissues during mouse and human leptospirosis.

### High expression of VCAM-1, E-selectin and P-selectin in tissues of *L*. *interrogans*-infected mice

**Fig 8:**
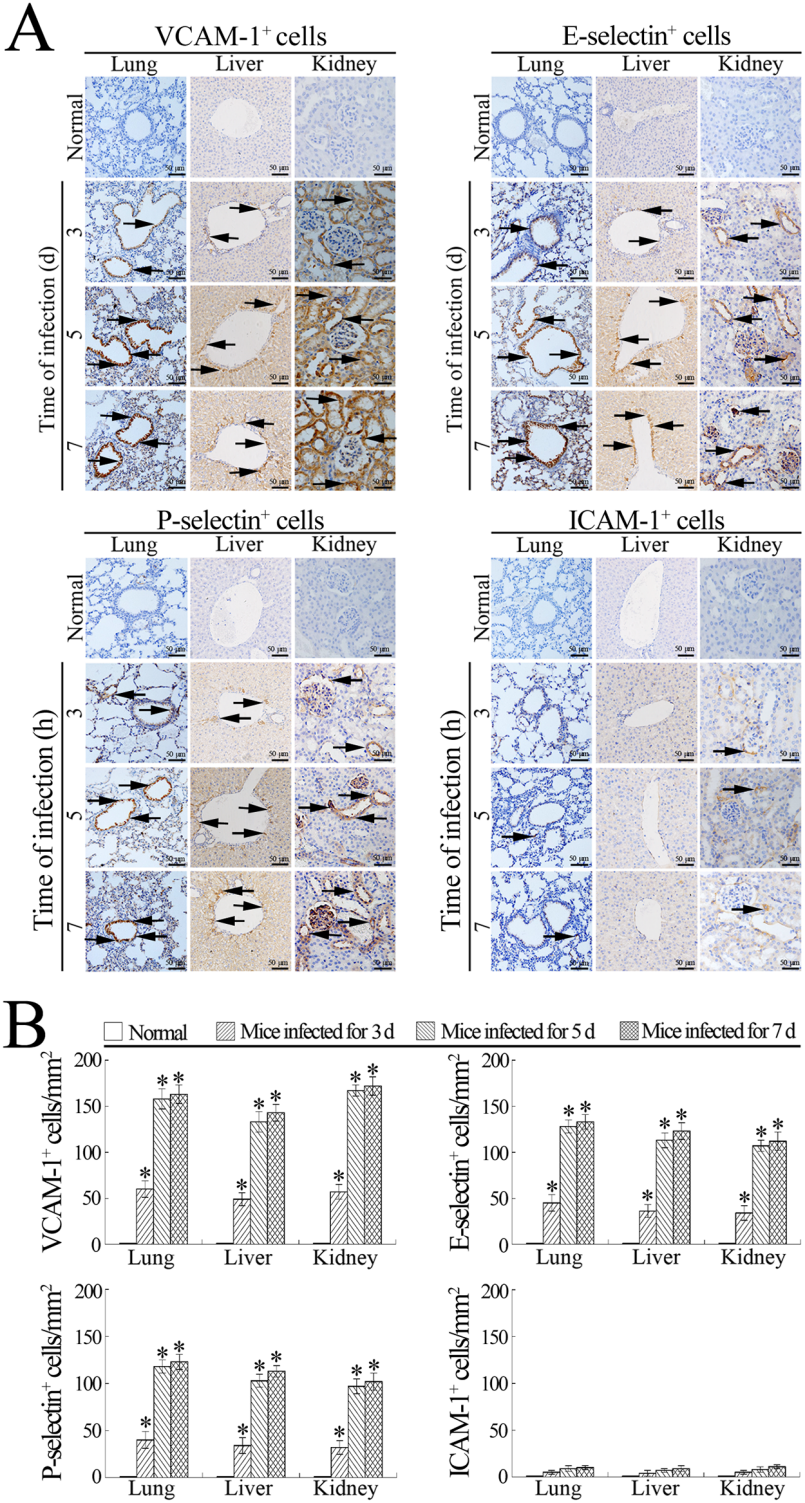
High expression of VCAM-1, P-selectin or E-selectin in tissues of *L*. *interrogans*- infected mice. **(A)** Expression of different adhesion molecules of mononuclear-macrophages and neutrophils in the lung, liver and kidney tissues from *L*. *interrogans*-infected C3H/HeJ mice, estimated by immunohistochemistry for the indicated infection times. The arrows indicate the expression of different adhesion molecules. **(B)** Expressed adhesion molecules in the tissues from *L*. *interrogans*-infected mice, estimated by analysis using Image-Pro Plus software. Statistical data from experiments such as shown in A. Bars show the means ± SD of three independent experiments. *: *p*<0.05 *vs* the normal animals.

The immunohistochemical examination showed that the expression of VCAM-1, a major adhesion molecule of macrophages, and E- and P-selectin, two adhesion molecules for both macrophages and neutrophils [19], were significantly up-regulated in the lung, liver and kidney tissues from *L*. *interrogans*-infected C3H/HeJ mice, but much lower expression of ICAM-1 (the efficiency of anti-mouse-ICAM-1-IgG as shown in S1 File and S2 Fig), a major adhesion molecule of neutrophils [19], could be found in the lung and kidney tissues (Fig 8A and 8B). The results suggested that the high levels of macrophage VECAMs are involved in the infiltration of mononuclear-macrophages from peripheral blood into infected tissues during leptospirosis.

## Discussion

Macrophages and neutrophils are central mediators of the host innate immune system and play a key role in phagocytosis and killing of invaded pathogens at early stages of microbial infection [49]. However, the two phagocytes also have distinctive functional properties during infection by different prokaryotic pathogens. Many pathogens, such as *Staphylococcus aureus* and *Pseudomonas aeruginosa*, are killed by neutrophils and cause a typical clinical symptom at the site of infection characterized by formation of pus, a mass of killed pathogens and dead neutrophils [50,51]. On the contrary, some bacterial pathogens, such as *Salmonella* species and *Mycobacterium tuberculosis*, are killed by mononuclear-macrophages and cause a nonpyogenic infection [18,52]. Leptospirosis is well known as a nonpyogenic infection, but the infiltrated phagocytic cell types had not been documented carefully. C3H/HeJ mice are TLR4 gene deficient, but more susceptible to *L*. *interrogans* and display typical histopathological changes of leptospirosis compared to other species of mice [39,40,53]. Moreover, TLR2 but not TLR4 is responsible for recognizing leptospiral lipopolysaccharide (LPS), the major inducer of inflammatory reactions and immune responses during leptospirosis [54,55]. In the present study, we confirmed that peripheral blood mononuclear-macrophages but not neutrophils are the main infiltrating phagocytes in all the examined internal organs from *L*. *interrogans*-infected mice, which could help to explain leptospirosis as a nonpyogenic infectious disease.

Chemokines and endothelial cell adhesion molecules directly mediate the migration of mononuclear-macrophages and neutrophils from peripheral blood to sites of infection [19]. The chemokines are grouped into CC, CXC, CX3C and XC subfamilies based on the position of their initial cysteine residues, which bind to and signal through seven transmembrane-spanning G-protein-linked receptors expressed on mononuclear-macrophages and neutropphil [56,57]. The members in the CC family such as CCL1/I-309, CCL2/MCP-1, CCL3/MIP-1α, CCL5/RANTES and CCL12/MCP-5 are the major chemokines of mononuclear-macrophages, while the members in CXC family such as CXCL1/KC, CXCL2/MIP-2 and CXCL5/LIX are mostly chemotactic for neutrophils [57]. The murine chemokines KC and MIP-2 are considered as the functional homologues of human IL-8, an important chemokine of human neutrophils [58]. In the migration process, both mononuclear-macrophages and neutrophils need to adhere to vascular endothelial cells, the former through combination of their β1-integrin VLA-4 with endothelial VCAM-1 and the latter through combination of their β2-integrin Mac-1 or LFA-1 with endothelial ICAM-1 [19,59]. E-selectin and P-selectin expressed by vascular endothelial cells also play a role in early stages of mononuclear-macrophage or neutrophil-endothelial cell interactions [19,57]. In the present study, we found that the five macrophage chemokines mentioned above but not the three neutrophil chemokines in sera from *L*. *interrogans*-infected mice and leptospirosis patients were significantly increased, while the VCAM-1, and E-selectin and P-selectin in the internal organ tissues of *L*. *interrogans*-infected mice, but not ICAM-1, were present at high expression levels. Moreover, the macrophage chemokines rather than neutrophil chemokines in the sera from leptospirosis patients were also notably increased. The results suggested that high levels of macrophage-specific chemokines and VECAMs may be the main mechanism responsible for peripheral blood mononuclear-macrophage infiltration during leptospirosis. The few neutrophils in the internal organ tissues of infected mice may be due to the high expression of E- and P-selectins, which can affect the two phagocyte cell types.

Previous histopathological studies revealed that mononuclear-macrophages but not neutrophils acted as the infiltrating cells in biopsy samples from leptospirosis patients [60,61]. Human neutrophils and mouse macrophages have been confirmed to enable killing non-pathogenic but not pathogenic *Leptospira* species [62,63]. However, until now the hypothesis that macrophages rather than neutrophils act as the major phagocytes that phagocytose and eliminate invading pathogenic *Leptospira* species in infected hosts has not been conclusively proven. Both macrophages and neutrophils kill invaded and internalized pathogens through several strategies, such as secretion of microbicidal granules and formation of extracellular traps, but phagocytosis and fusion of phagosomes and lysosomes are the major mechanism by which the two phagocytes eliminate pathogens [14,64–66]. Our previous study revealed that phagosomes harboring leptospires and lysosomes in macrophages from human origin co-localize less frequently than in macrophages from mouse origin, which may be responsible for long-term loading of pathogenic *Leptospira* species in the infected rodent animals and rapid pathogenesis in the infected humans [67]. In the present study, we also demonstrated that the ratios of phagosomes containing leptospires and lysosomes in human macrophages were noticeably lower than in murine macrophages. In particular, we also observed much lower *Leptospira*-phagocytosing and phagolysosome-forming ratios in the infected Hu- or Ms-neutrophils tha the Hu- or Ms-macrophages. Taken together with the high infiltration of peripheral blood mononuclear-macrophages into tissues of *L*. *interrogans*-infected mice described above, macrophages but not neutrophils acts appear to be the main phagocyte that eliminates invading *Leptospira* during leptospirosis.

After formation of phagolysosomes, phagocytes use several strategies to kill phagocytosed pathogens, such as low pH, various hydrolases, bactericidal peptides and ROS [64]. Except for the low pH and lysosomal hydrolases, intracellular ROS has been confirmed as an important agent to kill the pathogens in phagolysosomes through direct oxidization to inactivate and damage nucleic acids, proteins, lipids and carbohydrates [52]. NO can inhibit pathogens by reaction with microbial structural elements, metabolic enzymes and virulence-associated molecules [68]. High NO levels have been confirmed to play an important role in killing *Salmonella typhimurium* and *Vibrio cholerae* [69,70]. Intracellular free Ca^2+^ is a critical factor for activation of phagocytes such as inducing phagocytosis, the respiratory burst, maturation of phagosomes, and secretion of bactericidal substances [71]. Previous studies showed that high [Ca^2+^]i is involved in killing intracellular *Listeria monocytogenes* and *Helicobacter pylori* [71,72]. In the present study, we revealed that the total ROS and NO levels and [Ca^2+^]i in the *L*. *interrogans*-infected macrophages were markedly increased; and that inhibition of the total ROS, NO and [Ca^2+^]i changes could decrease significantly their leptospiral-killing ability. Conversely, the *L*. *interrogans*-infected neutrophils showed much lower ROS and NO levels and [Ca^2+^]i changes. The data suggested that high total ROS, NO and intracellular free Ca^2+^ levels are major factors used by macrophages to eliminate invading pathogenic *Leptospira* species. Since both human and mouse macrophages presented similar total ROS and NO levels and [Ca^2+^]i during infection, the lower leptospiral-killing ability of human macrophages than mouse macrophages may be due mainly to the lower numbers of phagosomes fusing with lysosomes.

## Supporting information

S1 File Supplementary materials. (DOC)

S1 Fig Efficiency of anti-mouse-CD11b or Ly6G-IgG. Efficiency of CD11b-IgG or Ly6G-IgG detecting Ms-macrophages or Ms-neutrophils, determined by immunohistochemical examination.
(TIF)

S2 Fig Efficiency of anti-mouse-ICAM-1-IgG. Efficiency of anti-mouse-ICAM-1-IgG detecting ICAM-1 in mouse spleen tissue, determined by immunohistochemical examination.
(TIF)
