# Correction: Mononuclear-macrophages but not neutrophils act as major infiltrating anti-leptospiral phagocytes during leptospirosis

**DOI:** 10.1371/journal.pone.0291717

**Published:** 2023-09-14

**Authors:** Xu Chen, Shi-Jun Li, David M. Ojcius, Ai-Hua Sun, Wei-Lin Hu, Xu’ai Lin, Jie Yan

During preparation of Fig 6A of this article [1], the “Normal/Lung” Mononuclear-macrophages panel was inadvertently duplicated and used to represent the “Normal/Lung” Neutrophils results. The updated Fig 6 provided with this notice presents the correct “Normal/Lung” Neutrophils results. The corresponding author stated that the corresponding results presented in Fig 6B were calculated using the correct images, and triplicate image data and individual level data underlying the Fig 6 results are provided in the S2 and S3 Files below respectively.

During the post-publication editorial assessment of this article, it was noted that the S1 File published with [1] did not include the data described in the article, but rather presented a summary of figure legends only. The corrected S1 File is provided below.

The Data Availability statement of this article [1] reads “All relevant data are within the paper and its Supporting Information files.” However, the individual-level data used to prepare the graphs presented in this article were not provided in the Supporting Information files. The individual level data underlying the graphs presented in this study are available in the S3–S9 Files provided with this notice.

**Fig 6:**
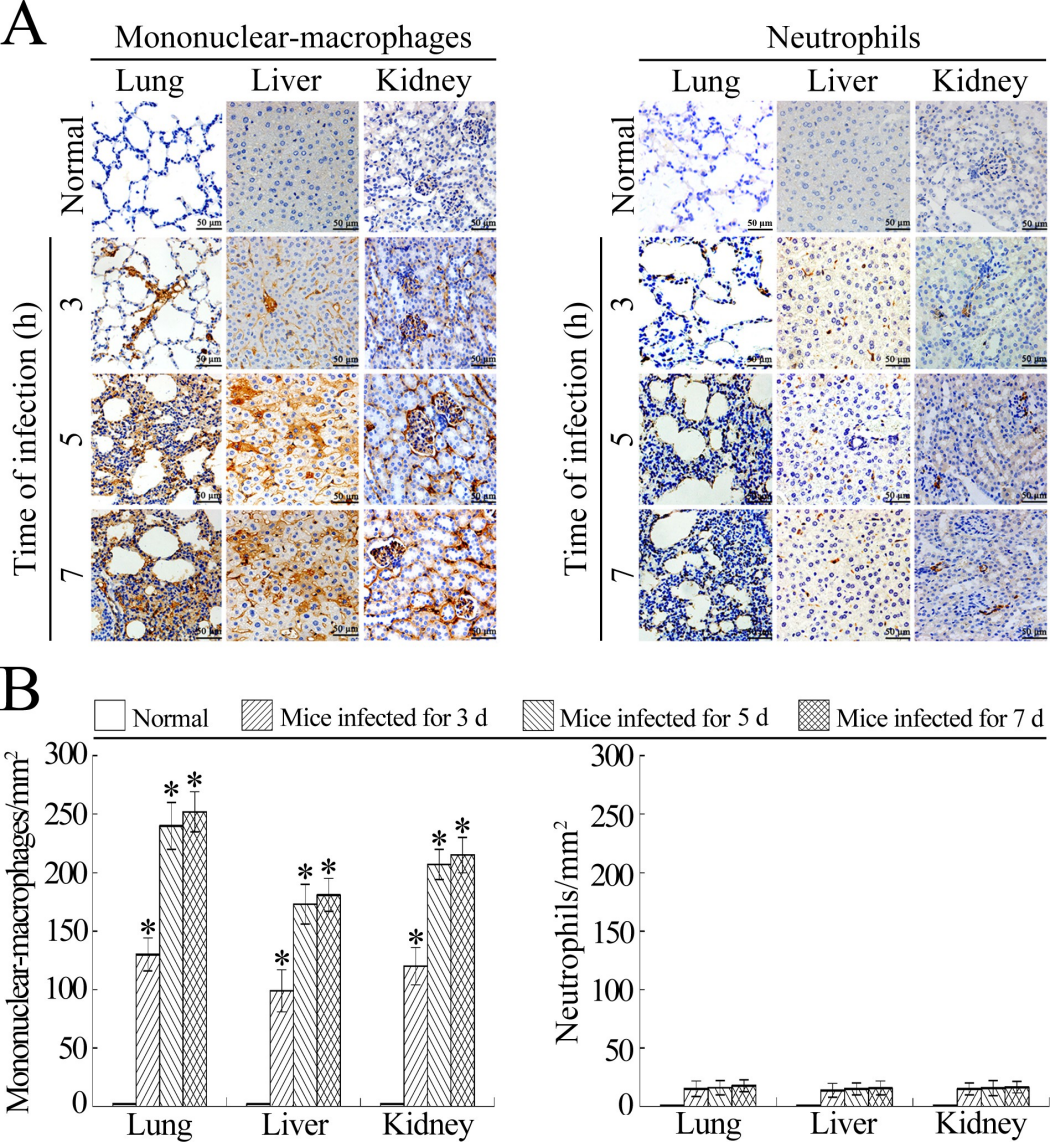
Mononuclear-macrophages from peripheral blood are the main infiltrating phagocytes during leptosirosis. **(A)** Infiltration of peripheral blood mononuclear-macrophages and neutrophils into the lung, liver and kidney tissues of *L*. *interrogans*-infected C3H/HeJ mice, visualized by immunohistochemistry for the indicated infection times. The mononuclear-macrophages or neutrophils were determined with CD11b or Ly6G antibody. **(B)** Infiltrated CD11b^+^ mononuclear-macrophages and Ly6G^+^ neutrophils in the lung, liver and kidney tissues from *L*. *interrogans*-infected mice, estimated by analysis using Image-Pro Plus software. Statistical data from experiments such as shown in A. Bars show the means ± SD of three independent experiments. *: *p*<0.05 vs the normal animals.

## Supporting information

S1 File Corrected version of the originally published S1 File. (DOC)

S2 File Triplicate image data underlying Fig 6 results. (PDF)

S3 File Individual level data underlying Fig 6 results. (XLS)

S4 File Individual level data underlying Fig 1 results. (ZIP)

S5 File Individual level data underlying Fig 2 results. (ZIP)

S6 File Individual level data underlying Fig 3 results. (ZIP)

S7 File Individual level data underlying Fig 4 results. (ZIP)

S8 File Individual level data underlying Fig 7 results. (ZIP)

S9 File Individual level data underlying Fig 8 results. (XLSX)
