# Supplementary material for: Mononuclear-macrophages but not neutrophils act as major infiltrating anti-leptospiral phagocytes during leptospirosis
Source: PLoS One. 2017 Jul 11;12(7):e0181014. doi: 10.1371/journal.pone.0181014 (PMC5507415; doi:10.1371/journal.pone.0181014)
Supplement: S1 File — (DOC) [file pone.0181014.s001.doc]

**Mononuclear-macrophages but not neutrophils act as major infiltrating anti-leptospiral phagocytes during leptospirosis**

Xu Chen^1,2,3¶^, Shi-Jun Li^4¶^, David M. Ojcius^5^, Ai-Hua Sun^6^, Wei-Lin Hu^1,2,3^, Xu’ai Lin^1,2,3*^, Jie Yan^1,2,3*^

**Supplementary Materials**

**High purity of the isolated primary Hu- or Ms-monocytes and neutrophil**s

The flow cytometric examination showed that the purity of isolated primary Hu- or Ms-monocytes was 97.4% or 97.7% while the purity of isolated primary Hu- or Ms-neutrophils was 98.9% or 98.5%.

**Low contamination of eosinophils and neutrophils in the isolated monocytes**

The flow cytometric examination showed that the ratios of contaminated eosinophils and neutrophils in the isolated Hu- or Ms-monocytes were 0.0% and 0.2%.or 0.0% and 0.1%.

**Low contamination of eosinophils and monocytes in the isolated neutrophils**

The flow cytometric examination showed that the ratios of contaminated eosinophils and monocytes in the isolated Hu- or Ms-neutrophils were 0.3% and 0.0%.or 0.4% and 0.1%.

**High efficiency of M-CSF inducing monocyte differentiation**

The flow cytmetric examination showed that 85.7% of the Hu-monocytes and 87.4% of the Ms-monocytes were differentiated into macrophages after induction with M-CSF.

**Efficiency of anti-mouse-CD11b or Ly6G-IgG**

The immunohistochemical examination showed that the rabbit anti-mouse-CD11b and rat anti-mouse-Ly6G-IgG could efficiently detect the Ms-macrophages and Ms-neutrophils, respectively (S1 Fig).

**Efficiency of anti-mouse-ICAM-1-IgG**

Mouse spleen tissue has been reported to express a high level of ICAM-1 [1,2]. The immunohistochemical examination showed that the rabbit anti-mouse-ICAM-1-IgG could efficiently detect the ICAM-1 expressed in mouse spleen tissue (S2 Fig).

**References**

1. Henninger DD, Panés J, Eppihimer M, Russell J, Gerritsen M, Anderson DC, et al. Cytokine-induced VCAM-1 and ICAM-1 expression in different organs of the mouse. J Immunol. 1997; 158:1825-1832.
2. Camacho SA., Heath WR, Carbone FR, Sarvetnick N, LeBon A, Karlsson L, et al. A key role for ICAM-1 in generating effector cells mediating inflammatory responses. Nat Immunol. 2001; 2(6):523-529.

**Figure Legend**

**S1 Fig.** Efficiency of CD11b-IgG or Ly6G-IgG detecting Ms-macrophages or Ms-neutrophils, determined by immunohistochemical examination.

**S2 Fig.** Efficiency of anti-mouse-ICAM-1-IgG detecting ICAM-1 in mouse spleen tissue, determined by immunohistochemical examination.
