# Supplementary figures and images for: Mononuclear-macrophages but not neutrophils act as major infiltrating anti-leptospiral phagocytes during leptospirosis
Source: PLoS One. 2017 Jul 11;12(7):e0181014. doi: 10.1371/journal.pone.0181014 (PMC5507415; doi:10.1371/journal.pone.0181014)

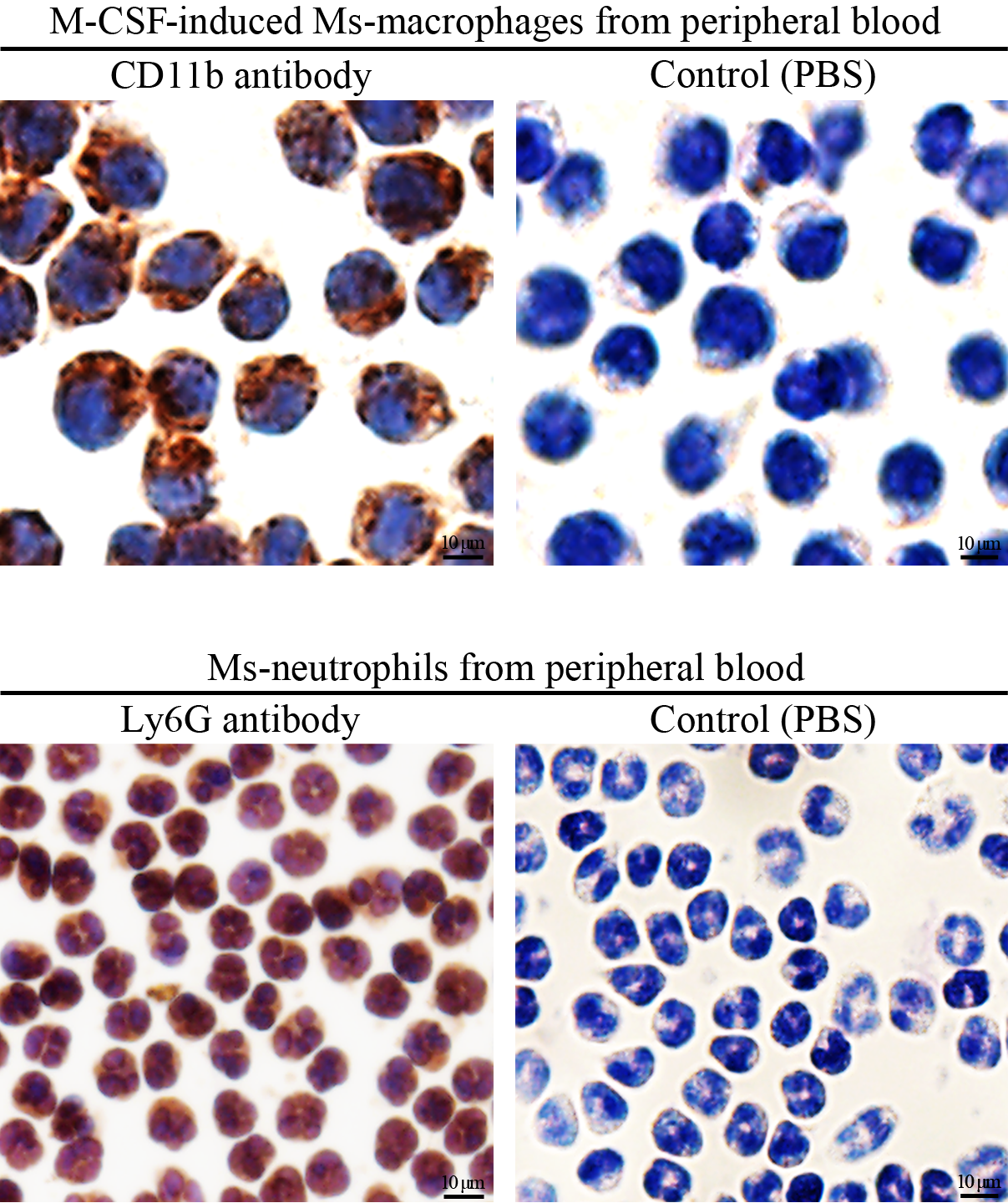

Supplement: S1 Fig — Efficiency of CD11b-IgG or Ly6G-IgG detecting Ms-macrophages or Ms-neutrophils, determined by immunohistochemical examination. (TIF) [file pone.0181014.s002.tif]

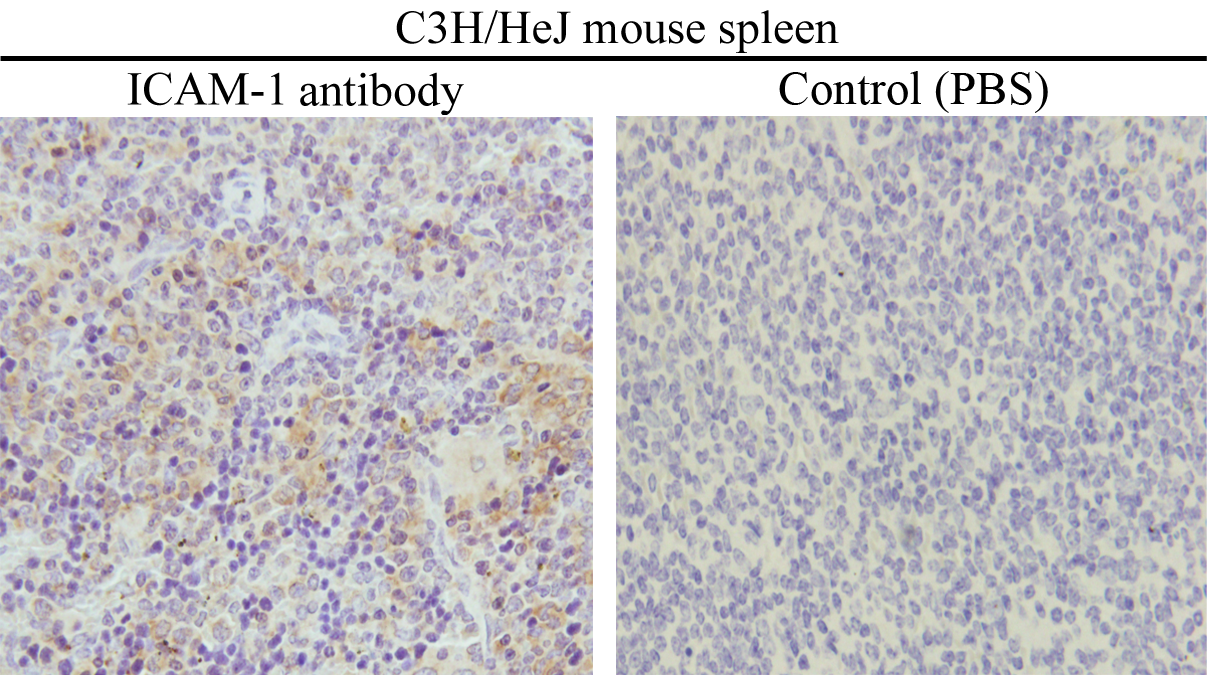

Supplement: S2 Fig — Efficiency of anti-mouse-ICAM-1-IgG detecting ICAM-1 in mouse spleen tissue, determined by immunohistochemical examination. (TIF) [file pone.0181014.s003.tif]
