# Supplementary material for: Correction: Mononuclear-macrophages but not neutrophils act as major infiltrating anti-leptospiral phagocytes during leptospirosis
Source: PLoS One. 2023 Sep 14;18(9):e0291717. doi: 10.1371/journal.pone.0291717 (PMC10501628; doi:10.1371/journal.pone.0291717)
Supplement: S1 File — (DOC) [file pone.0291717.s001.doc]

**Supplementary Materials File**

**Mononuclear-macrophages but not neutrophils act as major infiltrating anti-leptospiral phagocytes during leptospirosis**

Xu Chen^1,2,3☯^, Shi-Jun Li^4☯^, David M. Ojcius^5^, Ai-Hua Sun^6^, Wei-Lin Hu^1,2,3^, Xu’ai Lin^1,2,3*^, Jie Yan^1,2,3*^

**Results**

**High purity of the isolated primary Hu- or Ms-monocytes and neutrophils**


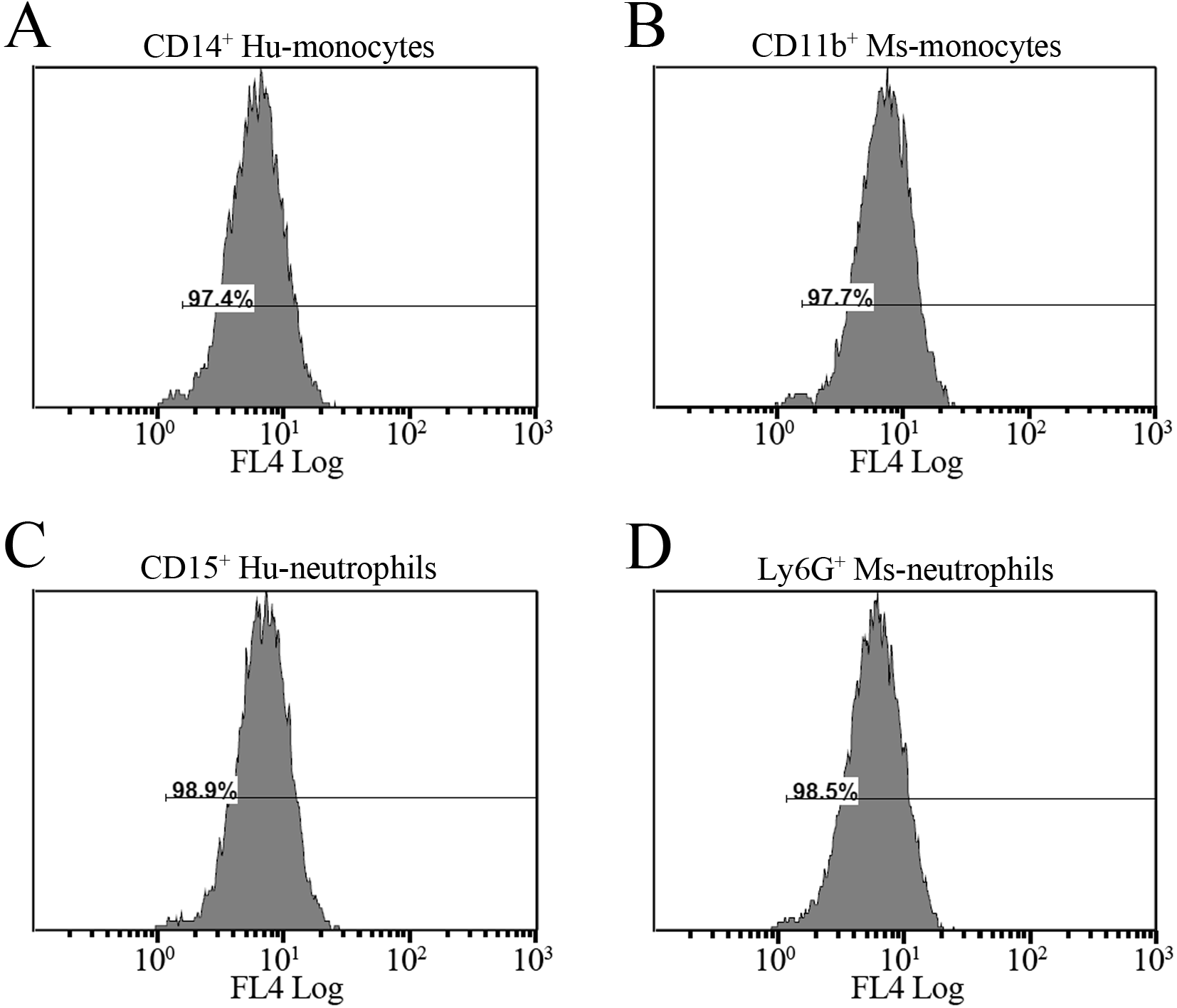


**Fig S1.** The flow cytometric examination showed that the purity of isolated primary Hu- or Ms-monocytes was 97.4% or 97.7%, while the purity of isolated primary Hu- or Ms-neutrophils was 98.9% or 98.5%.

**Low contamination of eosinophils and neutrophils in the isolated monocytes**


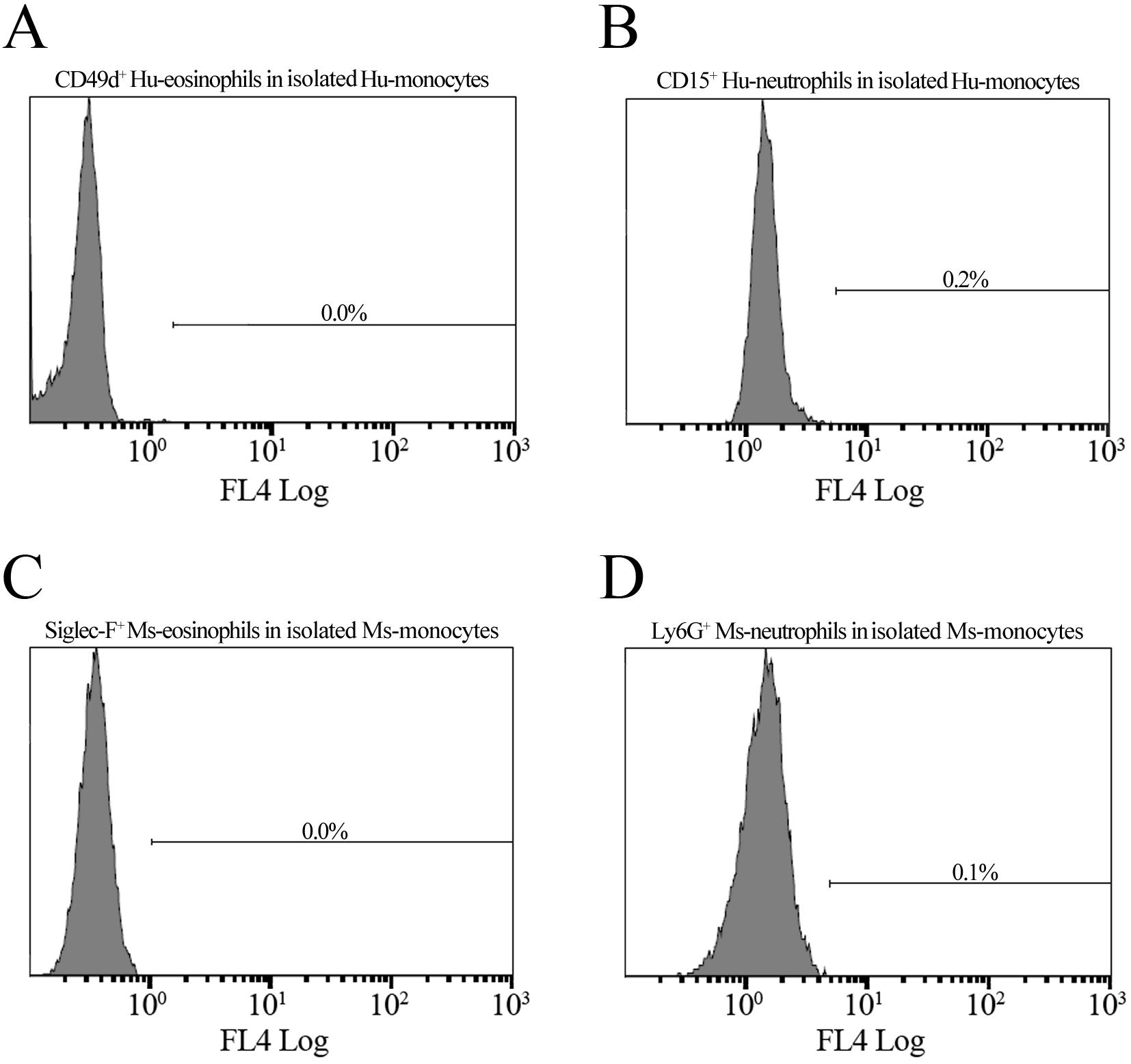


**Fig S2.**The flow cytometric examination showed that the ratios of contaminated eosinophils and neutrophils in the isolated Hu- or Ms-monocytes were 0.0% to 0.2%.

**Low contamination of eosinophils and monocytes in the isolated neutrophils**


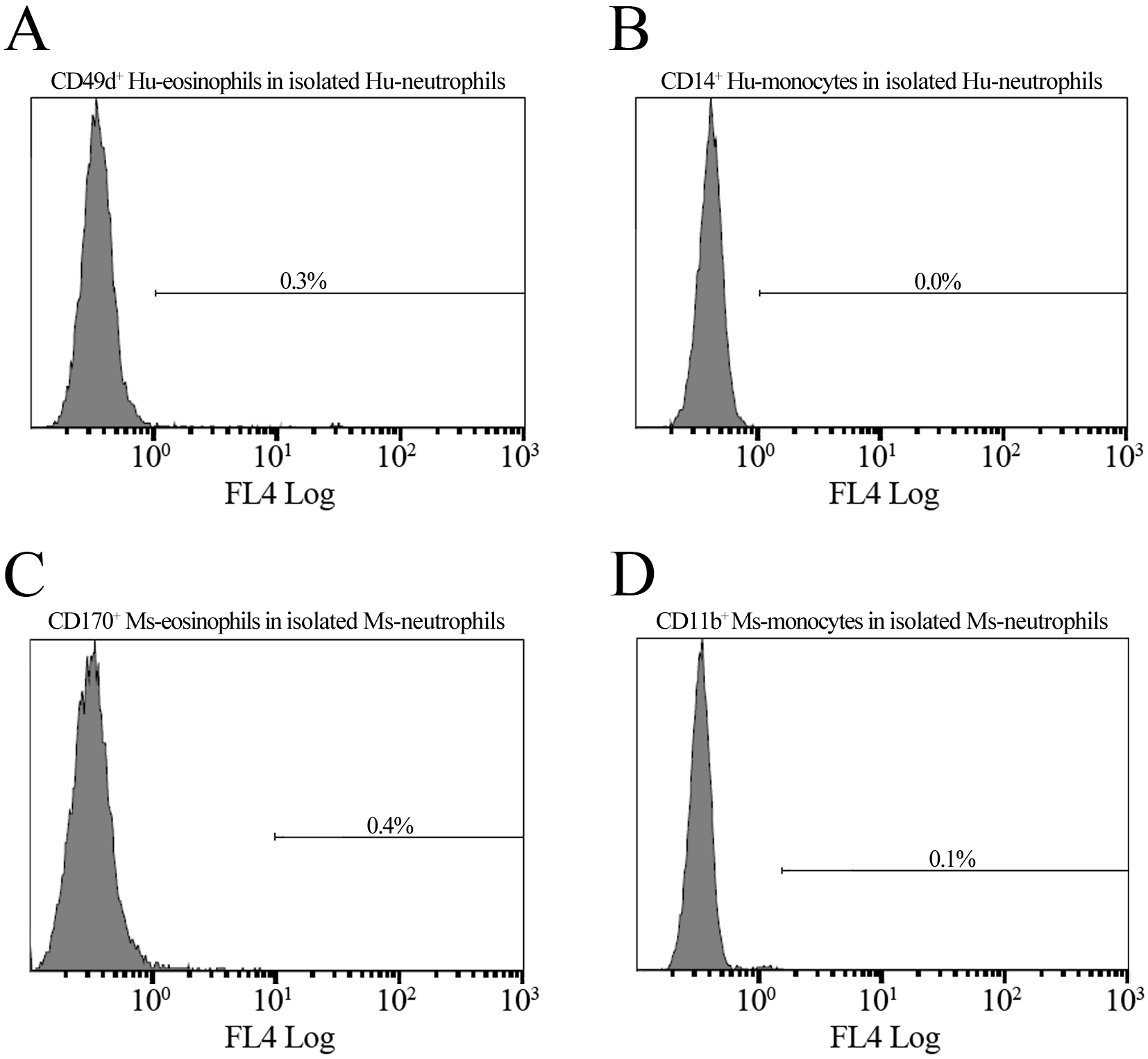


**Fig S3.** The flow cytometric examination showed that the ratios of contaminated eosinophils and monocytes in the isolated Hu- or Ms-neutrophils were 0.0% to 0.4%.

**High efficiency of M-CSF inducing monocyte differentiation**


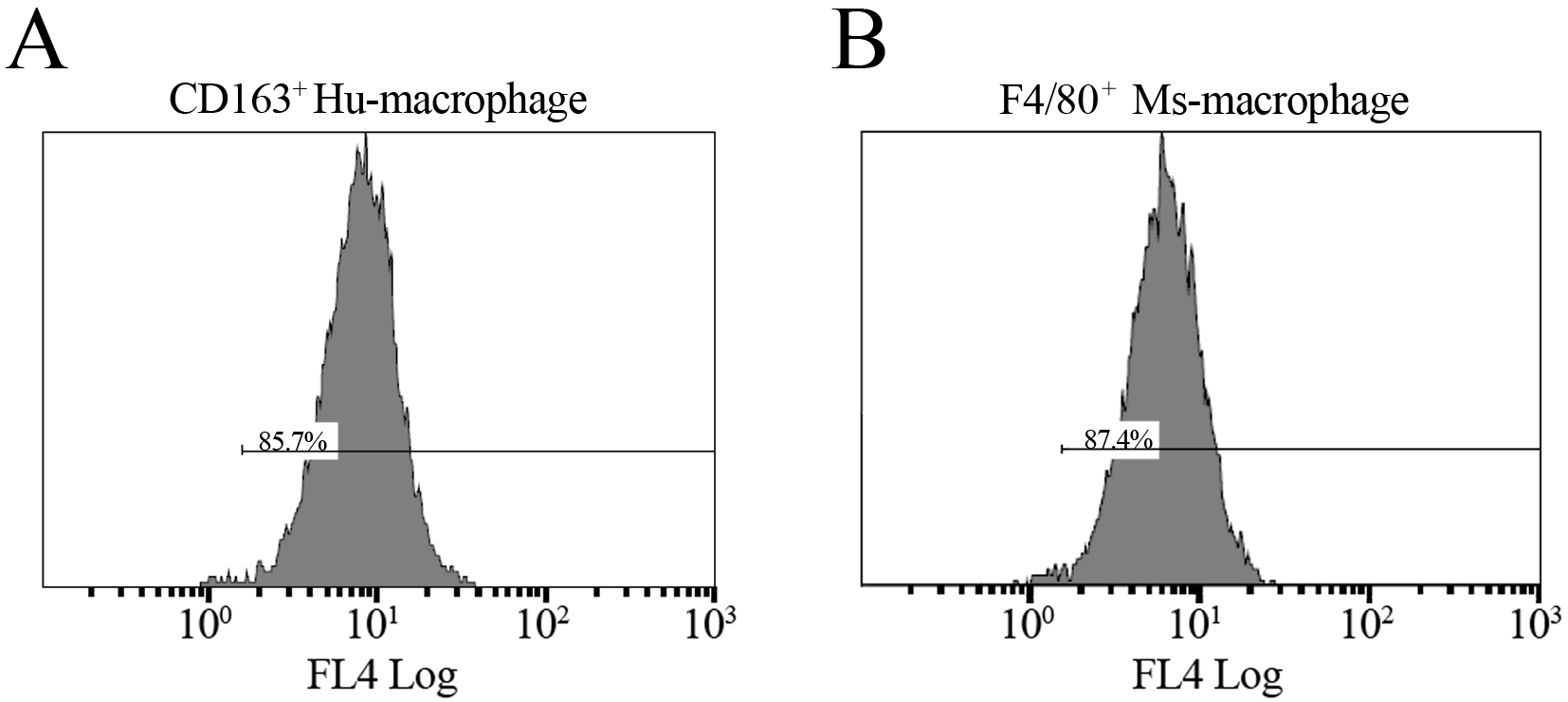


**Fig S4.** The flow cytmetric examination showed that 85.7% of the Hu-monocytes and 87.4% of the Ms-monocytes were differentiated into macrophages after induction with M-CSF.

**Efficiency of anti-mouse-CD11b or Ly6G-IgG**


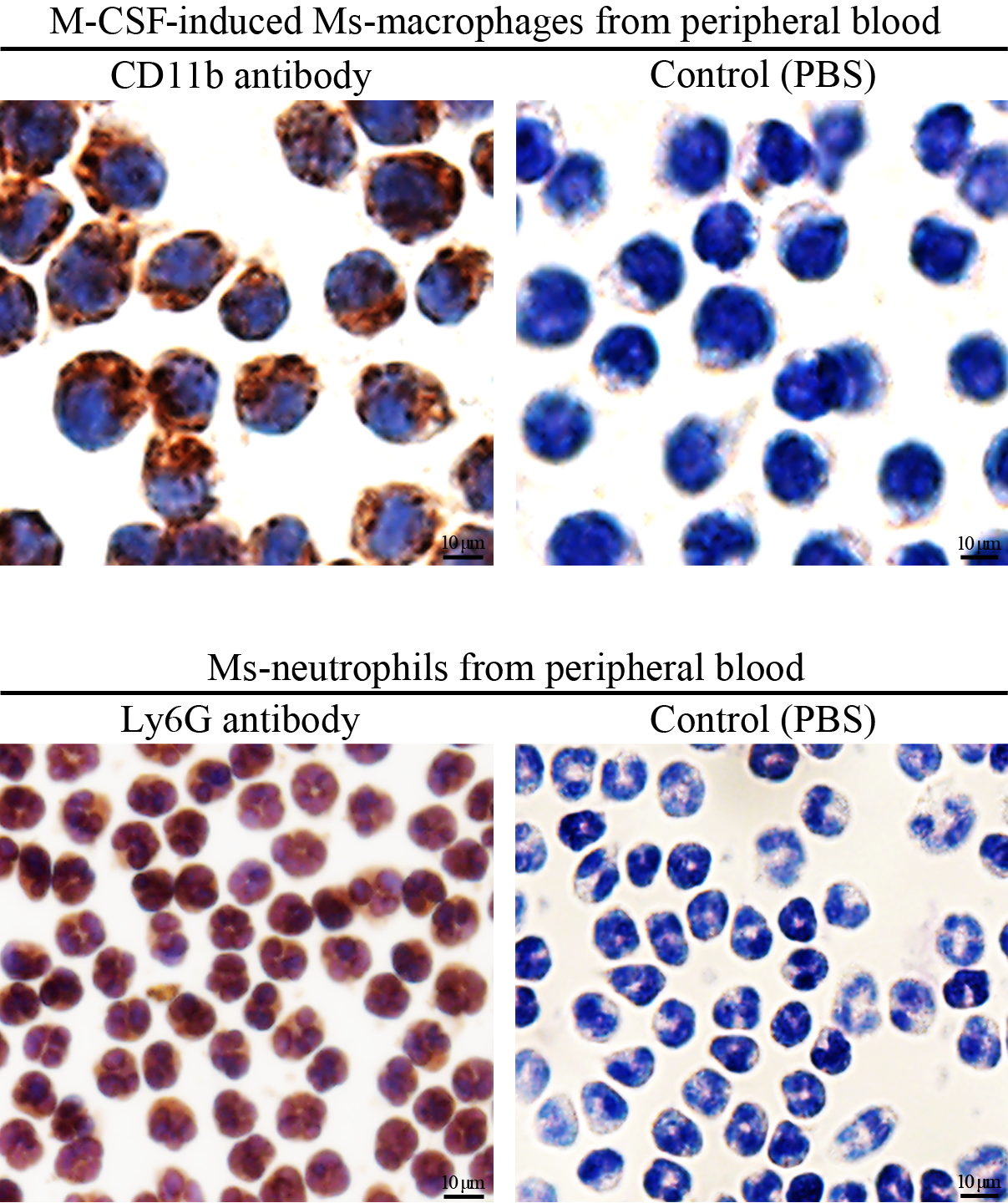


**Fig S5.** The immunohistochemical examination showed that the rabbit anti-mouse-CD11b and rat anti-mouse-Ly6G-IgG could efficiently detect the Ms-macrophages and Ms-neutrophils, respectively.

**Efficiency of anti-mouse-ICAM-1-IgG**


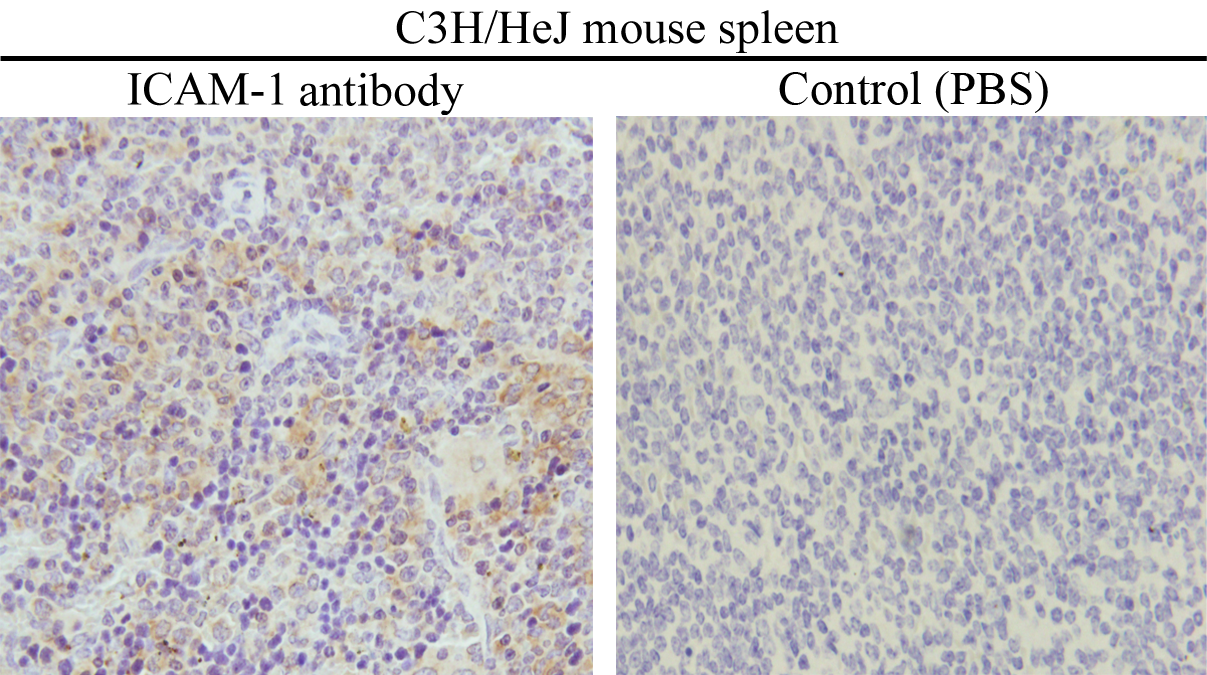


**Fig S6.** Mouse spleen tissue has been reported to express a high level of ICAM-1^[1,2]^. The immunohistochemical examination showed that the rabbit anti-mouse-ICAM-1-IgG could efficiently detect the ICAM-1 expressed in mouse spleen tissue.

**Reference**

1. Henninger DD, Panés J, Eppihimer M, Russell J, Gerritsen M, Anderson DC, et al. Cytokine-induced VCAM-1 and ICAM-1 expression in different organs of the mouse. J Immunol. 1997; 158:1825-1832.
2. Camacho SA., Heath WR, Carbone FR, Sarvetnick N, LeBon A, Karlsson L, et al. A key role for ICAM-1 in generating effector cells mediating inflammatory responses. Nat Immunol. 2001; 2(6):523-529.
