# Supplementary material for: Correction: Mononuclear-macrophages but not neutrophils act as major infiltrating anti-leptospiral phagocytes during leptospirosis
Source: PLoS One. 2023 Sep 14;18(9):e0291717. doi: 10.1371/journal.pone.0291717 (PMC10501628; doi:10.1371/journal.pone.0291717)
Supplement: S2 File — (PDF) [file pone.0291717.s002.pdf]

Triplicate image data for each Figure 6A panel (image data of CD11b$^+$ mononuclear-macrophages and Ly6G$^+$ neutrophils infiltration)

The image data of CD11b$^+$ mononuclear-macrophages infiltration

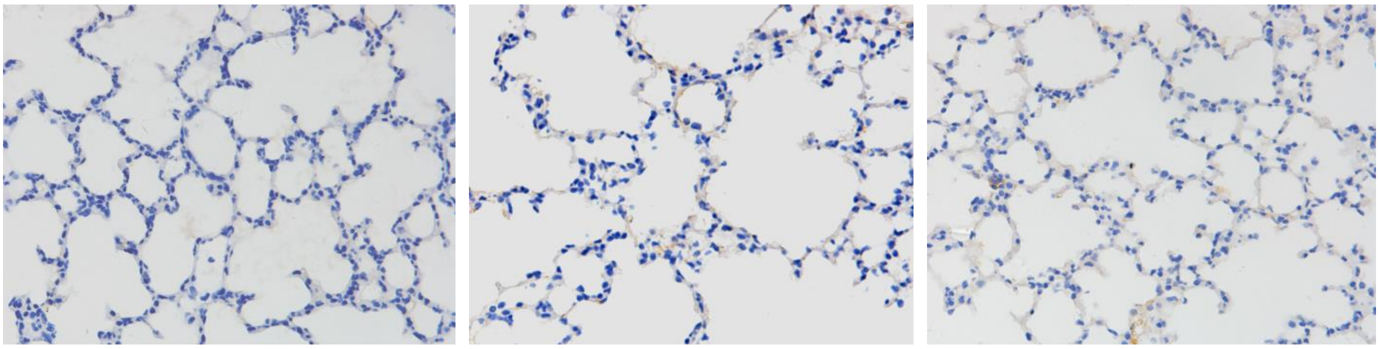

Test 1 Test 2 Test 3

Normal lung tissue

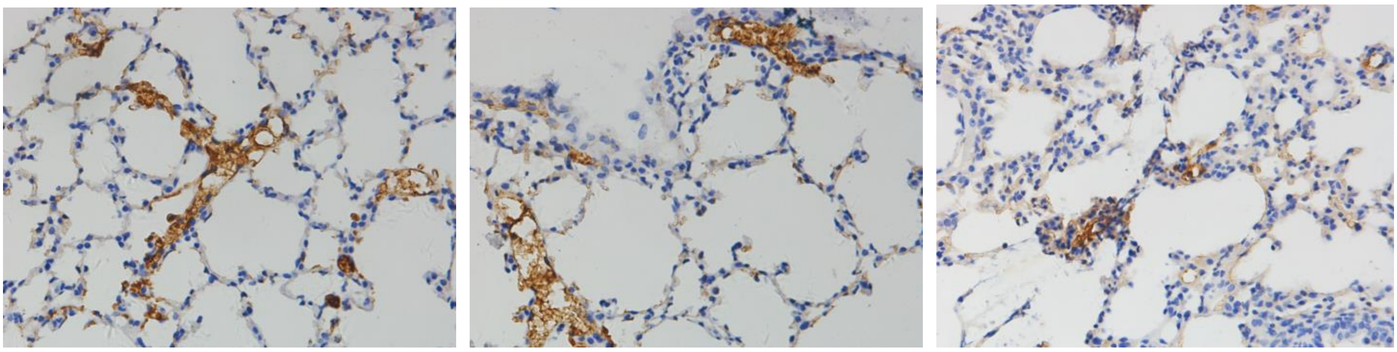

Test 1 Test 2 Test 3

Lung tissue for 3 days infection

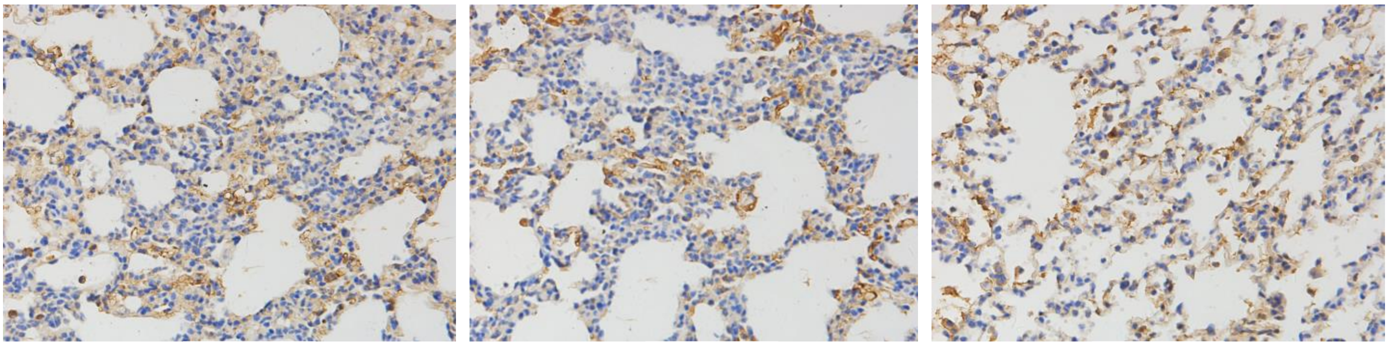

Test 1 Test 2 Test 3

Lung tissue for 5 days infection

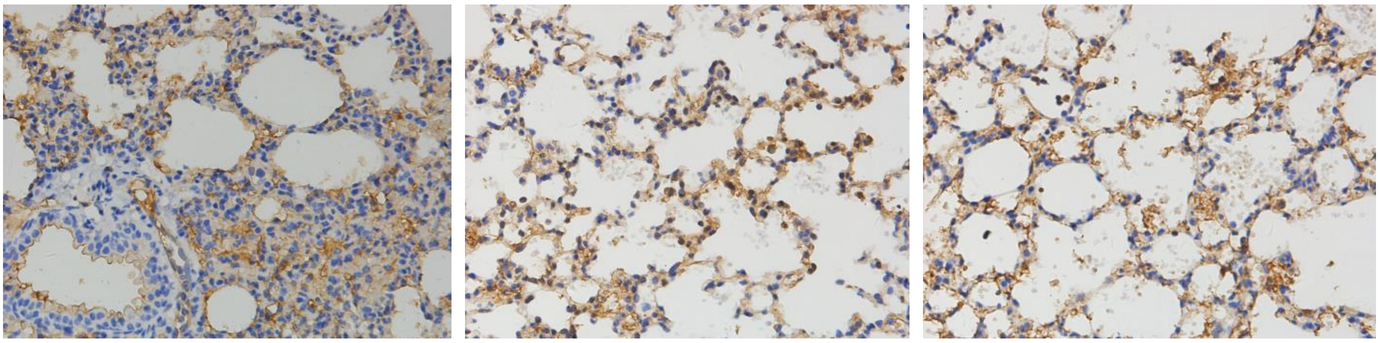

Test 1 Test 2 Test 3

Lung tissue for 7 days infection

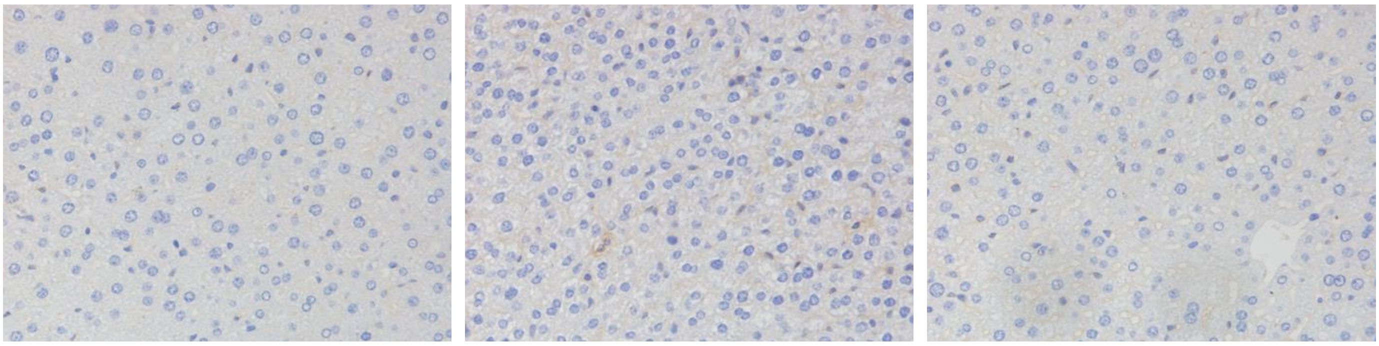

Test 1 Test 2 Test 2

Normal liver tissue

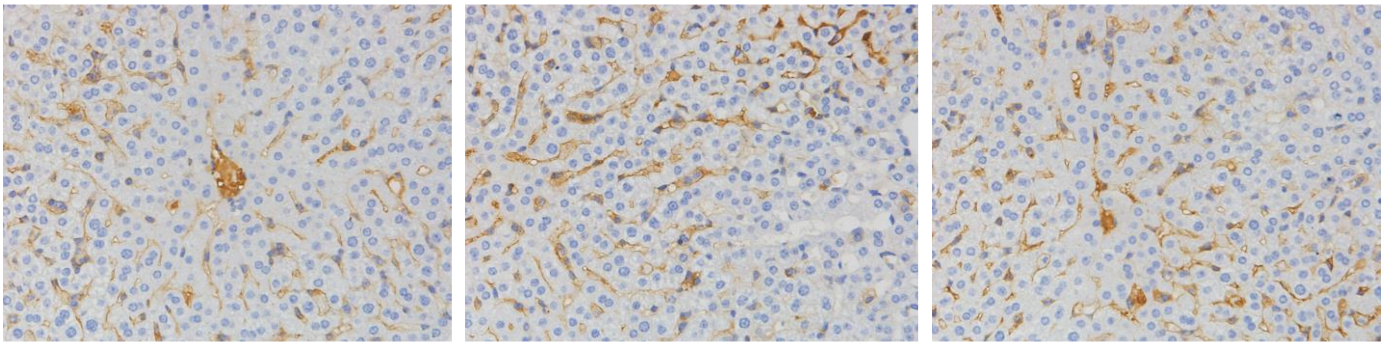

Test 1 Test 2 Test 3

Liver tissue for 3 days infection

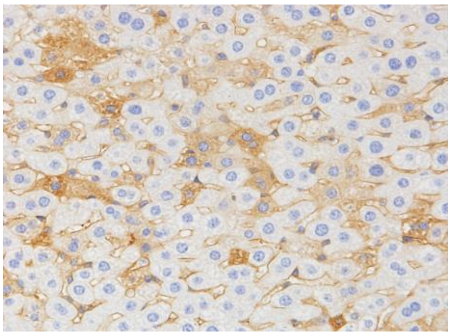

Test 1

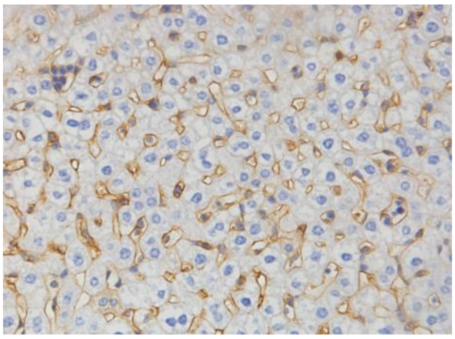

Test 2

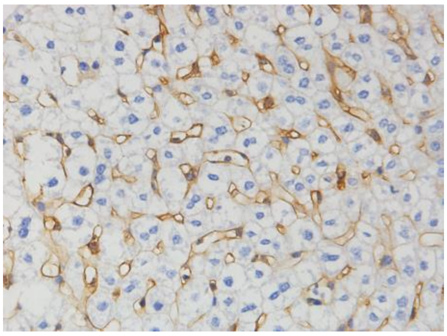

Test 3

Liver tissue for 5 days infection

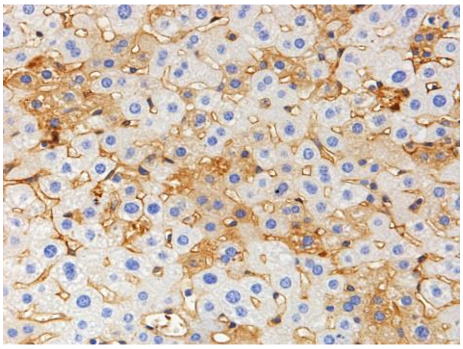

Test 1

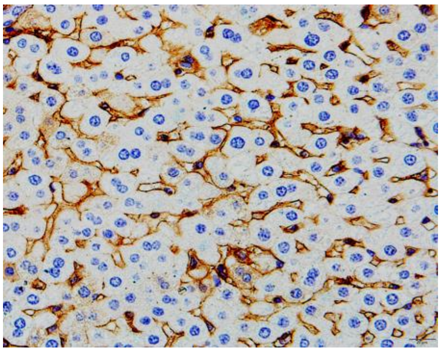

Test 2

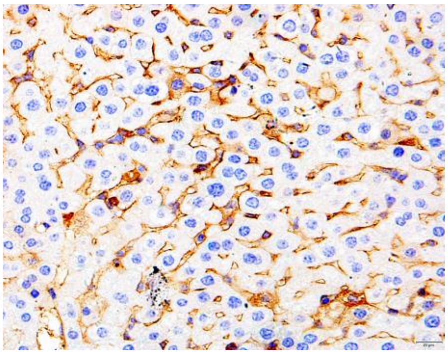

Test 3

Liver tissue for 7 days infection

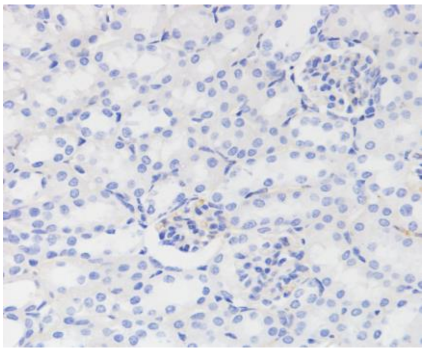

Test 1

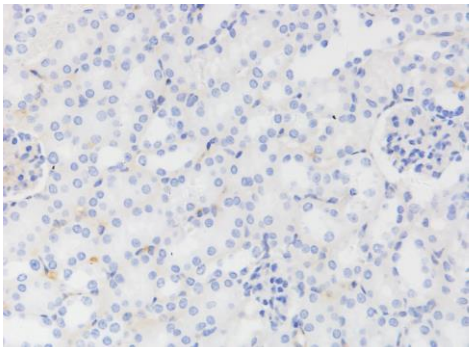

Test 2

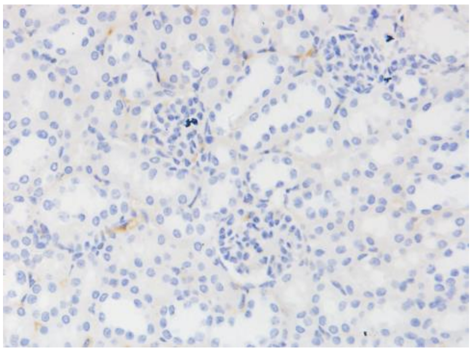

Test 3

Normal kidney tissue

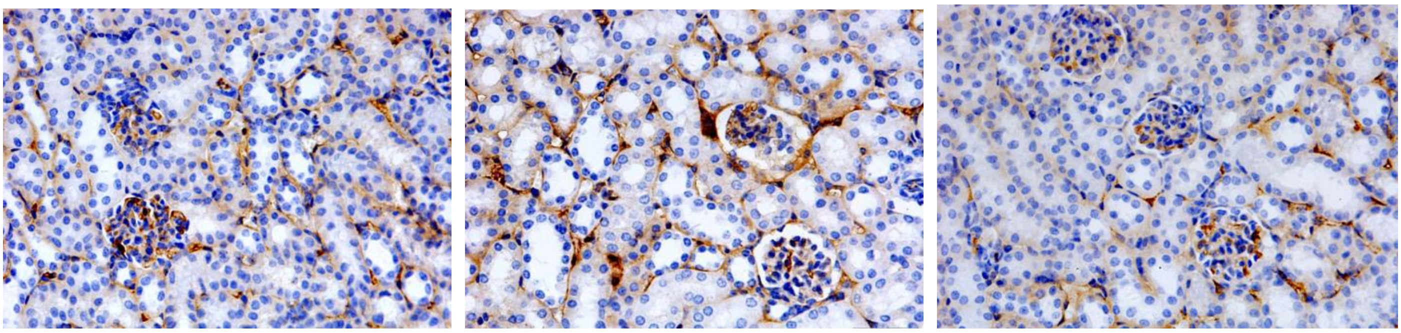

Test 1 Test 2 Test 3

Kidney tissue for 3 days infection

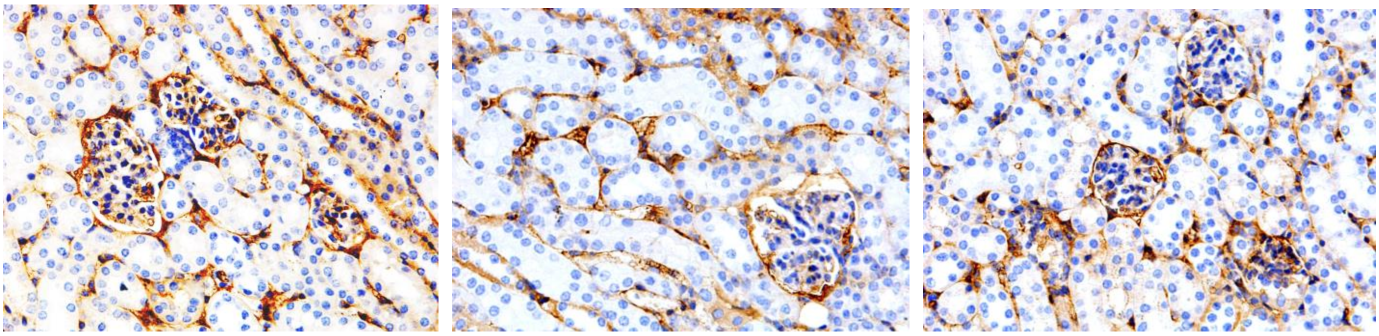

Test 1 Test 2 Test 3

Kidney tissue for 5 days infection

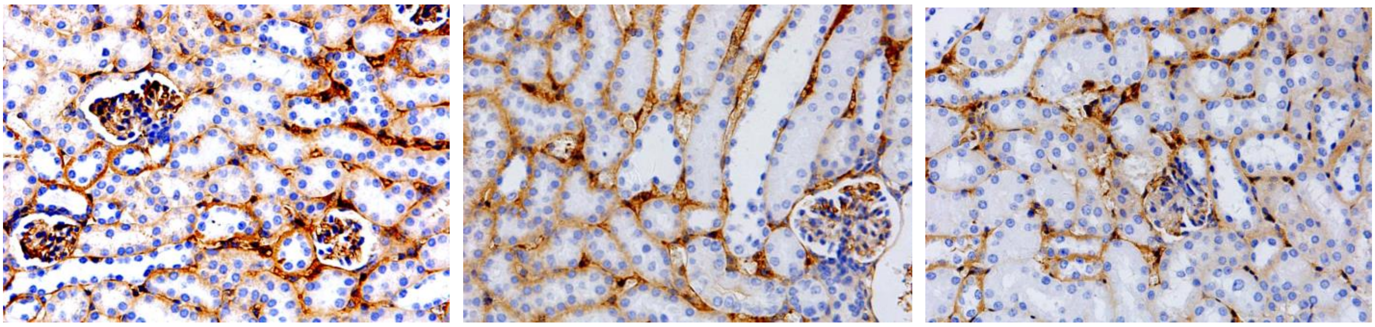

Test 1 Test 2 Test 3

Kidney tissue for 7 days infection

The image data of Ly6G^{+} neutrophils infiltration

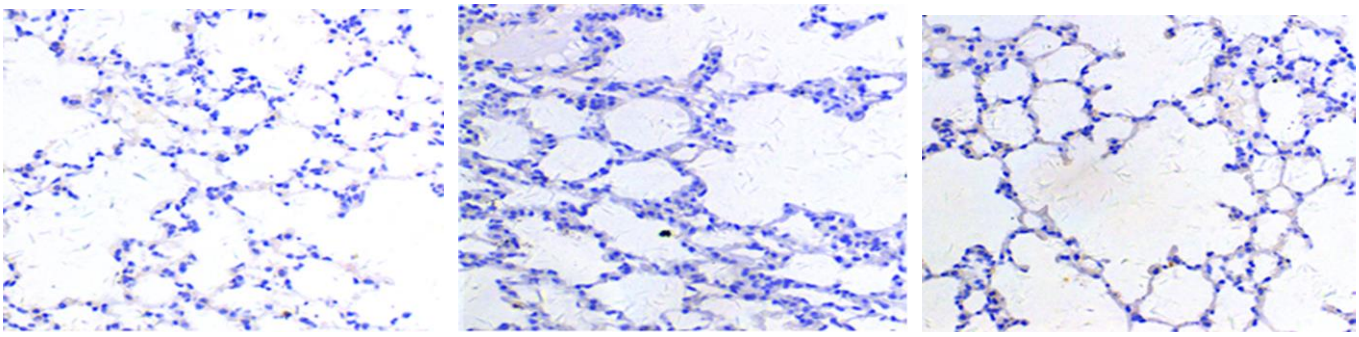

Test 1 Test 2 Test 3

Normal lung tissue

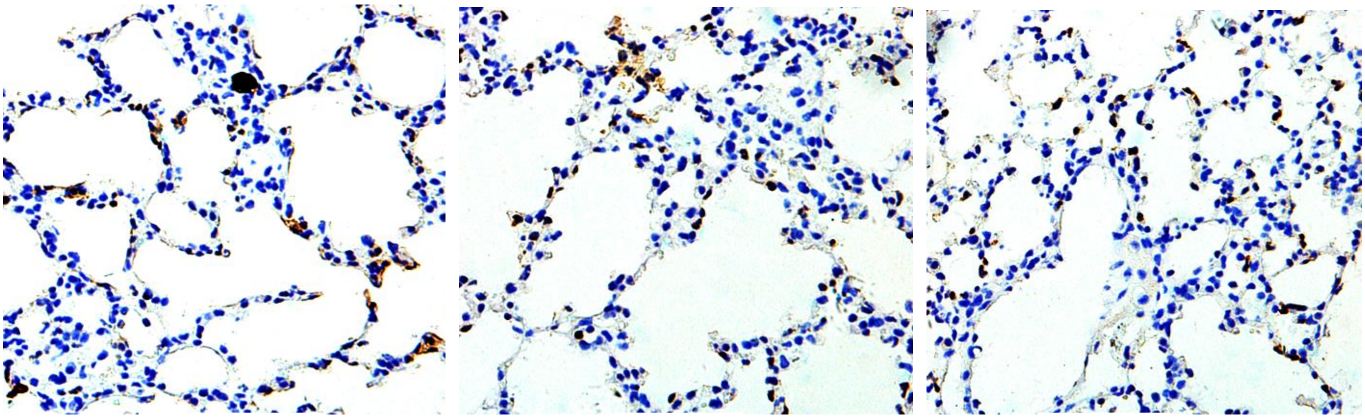

Test 1 Test 2 Test 3

Lung tissue for 3 days infection

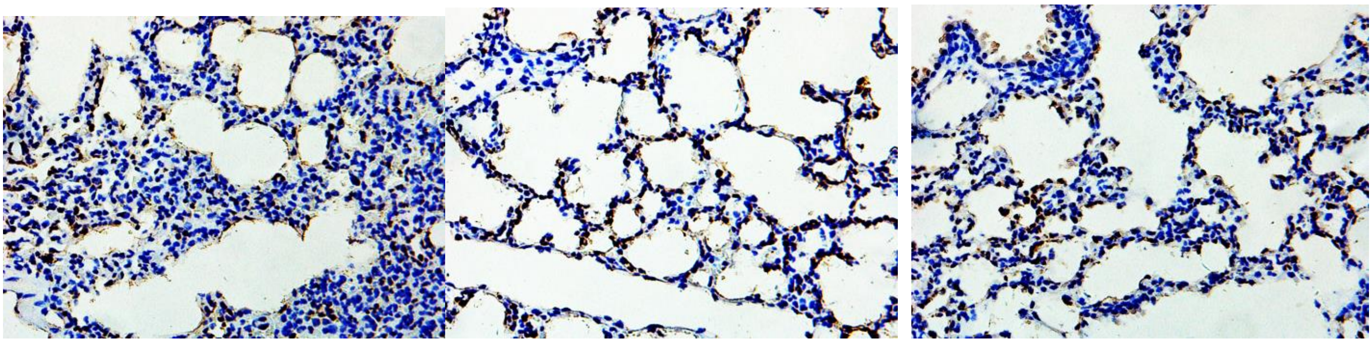

Test 1 Test 2 Test 3

Lung tissue for 5 days infection

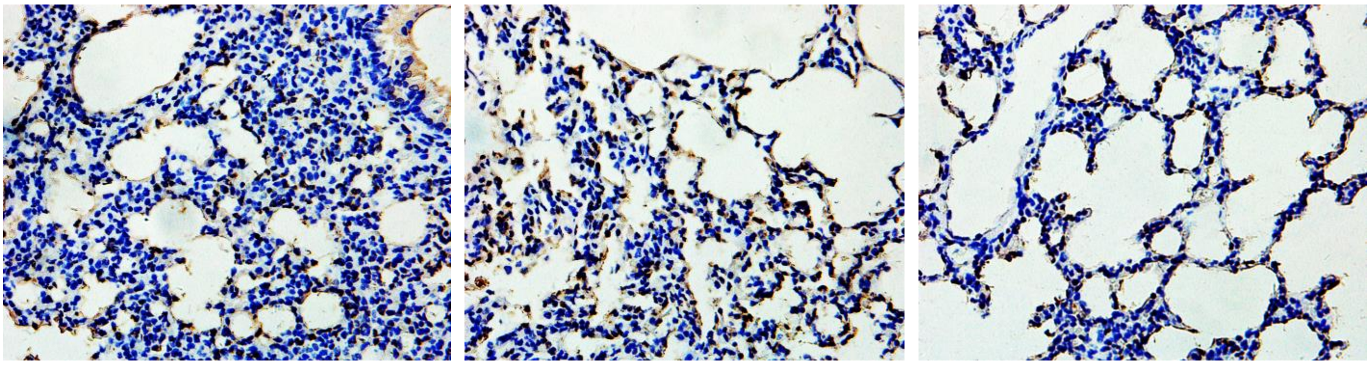

Test 1 Test 2 Test 3

Lung tissue for 7 days infection

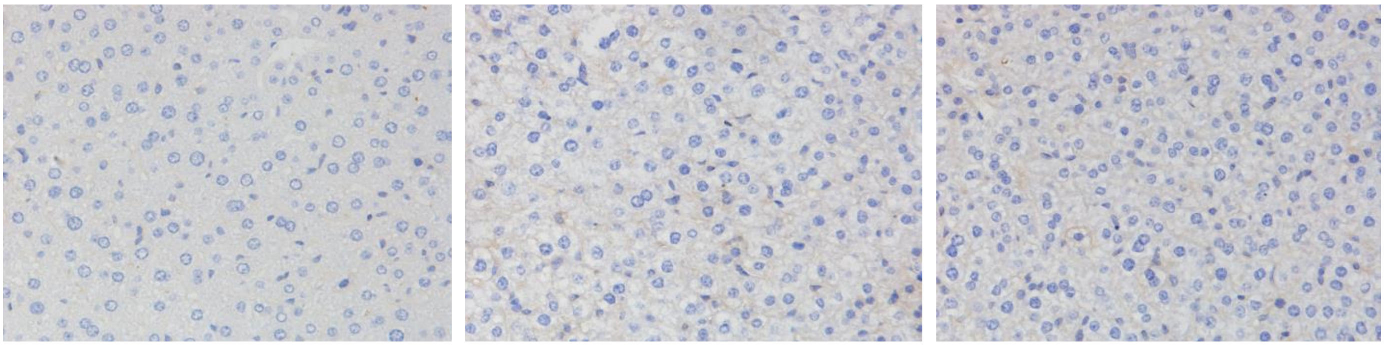

Test 1 Test 2 Test 3

Normal liver tissue

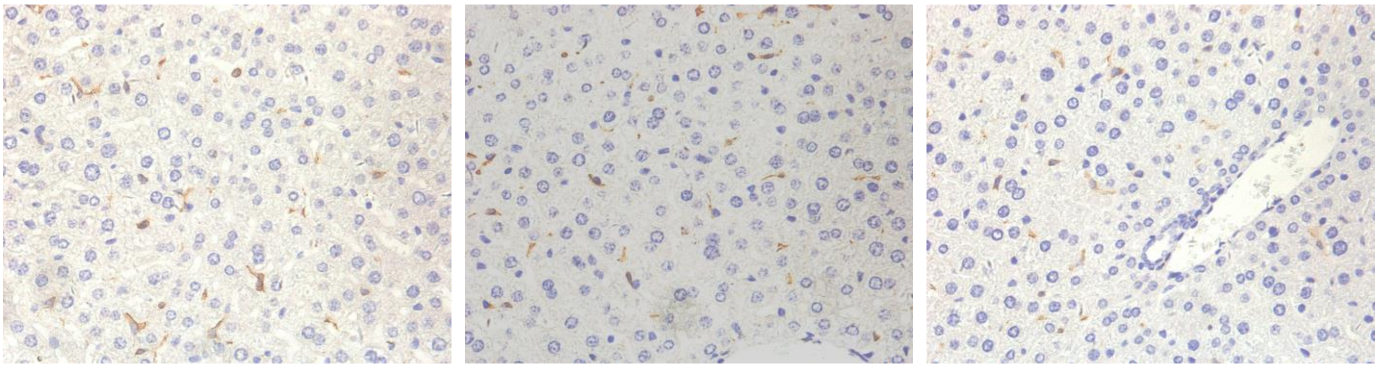

Test 1 Test 2 Test 3

Liver tissue for 3 days infection

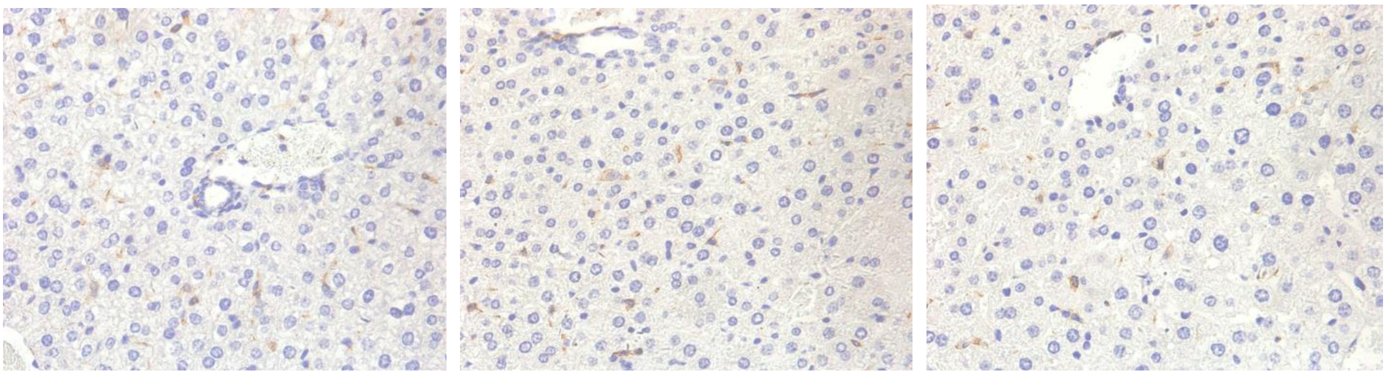

Test 1 Test 2 Test 3

Liver tissue for 5 days infection

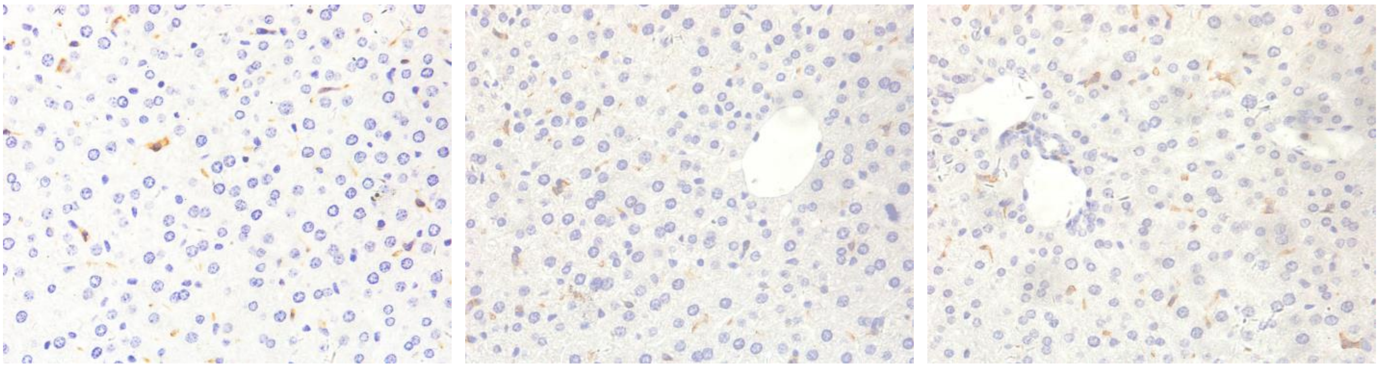

Test 1 Test 2 Test 3

Liver tissue for 7 days infection

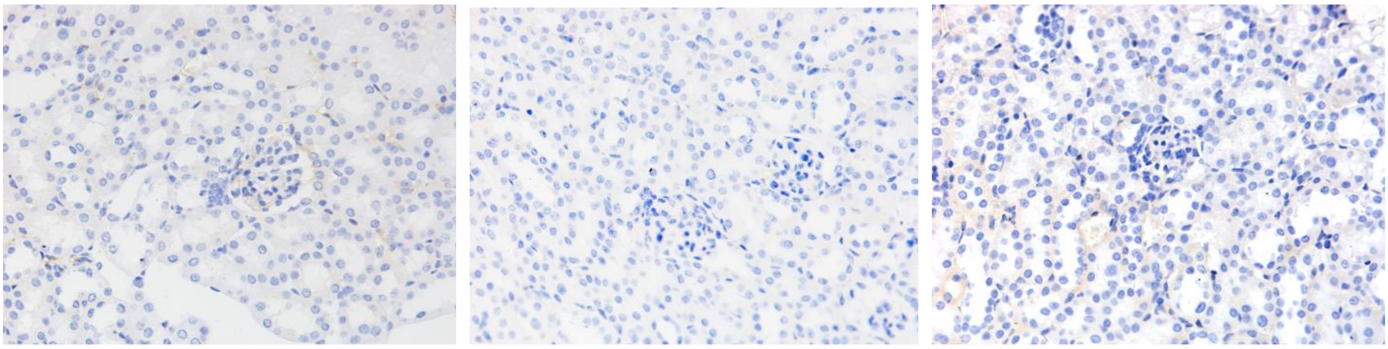

Test 1 Test 2 Test 2

Normal kidney tissue

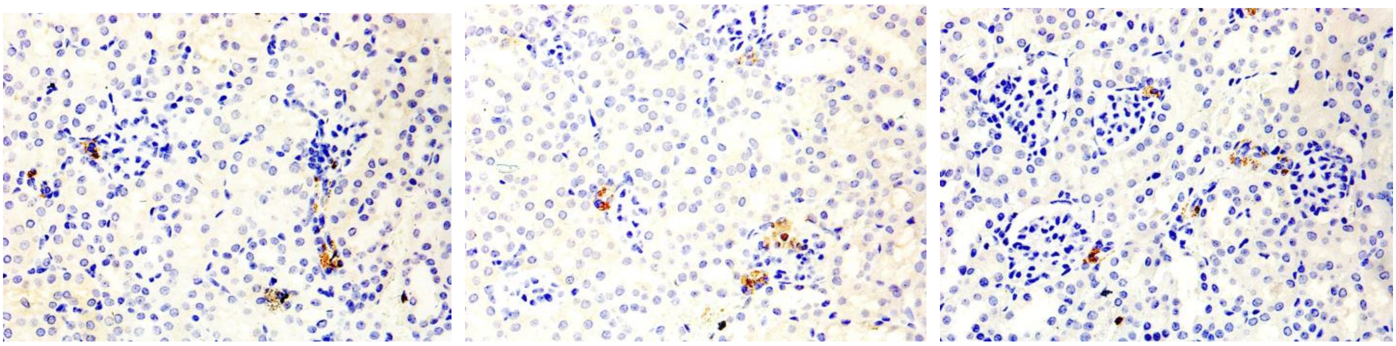

Test 1 | Test 2 | Test 3

Kidney tissue for 3 days infection

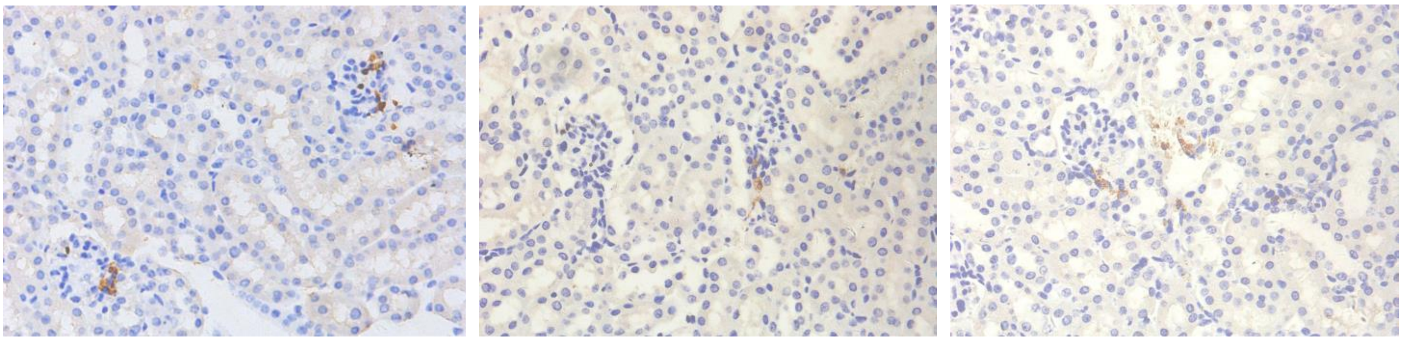

Test 1 | Test 2 | Test 3

Kidney tissue for 5 days infection

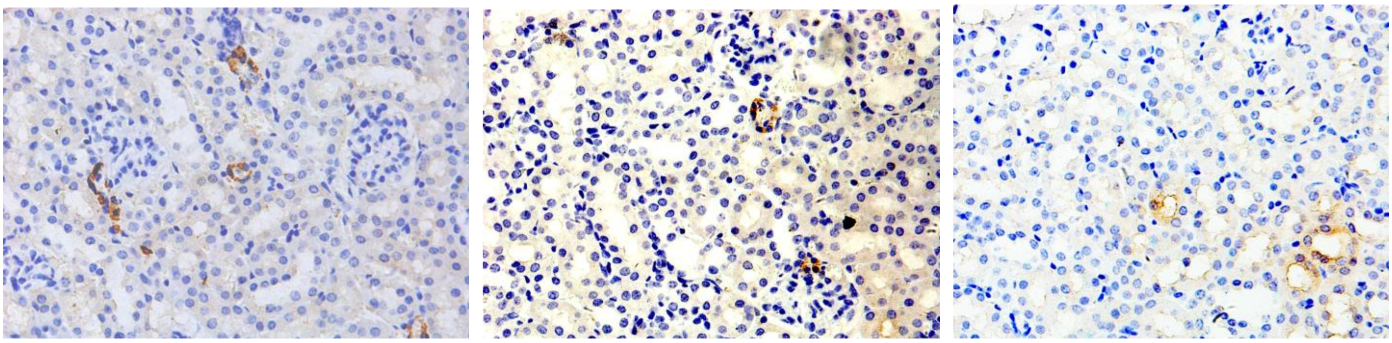

Test 1 | Test 2 | Test 3

Kidney tissue for 7 days infection
